# *De novo* design of energy transfer proteins housing excitonically coupled chlorophyll special pairs

**DOI:** 10.21203/rs.3.rs-2736786/v1

**Published:** 2023-04-21

**Authors:** Nathan Ennist, Shunzhi Wang, Madison Kennedy, Mariano Curti, George Sutherland, Cvetelin Vasilev, Rachel Redler, Valentin Maffeis, Saeed Shareef, Anthony Sica, Ash Hua, Arundhati Deshmukh, Adam Moyer, Derrick Hicks, Avi Swartz, Ralph Cacho, Nathan Novy, Asim Bera, Alex Kang, Banumathi Sankaran, Matthew Johnson, Mike Reppert, Damian Ekiert, Gira Bhabha, Lance Stewart, Justin Caram, Barry Stoddard, Elisabet Romero, C. Neil Hunter, David Baker

**Affiliations:** University of Washington; University of Washington; University of Washington; Institute of Chemical Research of Catalonia (ICIQ-CERCA); University of Sheffield; University of Sheffield; New York University School of Medicine; Institute of Chemical Research of Catalonia (ICIQ-CERCA); Institute of Chemical Research of Catalonia (ICIQ-CERCA); University of California, Los Angeles; University of California, Los Angeles; University of California, Los Angeles; University of Washington; University of Washington; University of Washington; University of Washington; University of Washington; Institute for Protein Design; University of Washington; Berkeley Center for Structural Biology; University of Sheffield; Purdue University; NYU School of Medicine; New York University School of Medicine; The Institute for Protein Design; University of California, Los Angeles; Fred Hutchinson Cancer Research Center; Institute of Chemical Research of Catalonia (ICIQ-CERCA); University of Sheffield; University of Washington

## Abstract

Natural photosystems couple light harvesting to charge separation using a “special pair” of chlorophyll molecules that accepts excitation energy from the antenna and initiates an electron-transfer cascade. To investigate the photophysics of special pairs independent of complexities of native photosynthetic proteins, and as a first step towards synthetic photosystems for new energy conversion technologies, we designed C_2_-symmetric proteins that precisely position chlorophyll dimers. X-ray crystallography shows that one designed protein binds two chlorophylls in a binding orientation matching native special pairs, while a second positions them in a previously unseen geometry. Spectroscopy reveals excitonic coupling, and fluorescence lifetime imaging demonstrates energy transfer. We designed special pair proteins to assemble into 24-chlorophyll octahedral nanocages; the design model and cryo-EM structure are nearly identical. The design accuracy and energy transfer function of these special pair proteins suggest that *de novo* design of artificial photosynthetic systems is within reach of current computational methods.

## Introduction

Photosynthetic proteins manipulate the distances and angles between chlorophyll (Chl) molecules to tune excitonic coupling and control absorption and fluorescence spectra, excited state dynamics, energy transfer, and electron tunneling (Croce & van Amerongen, 2014; Mirkovic et al., 2017; Romero et al., 2017; Şener et al., 2011). This control enables light harvesting and charge separation with quantum yields of 97% or higher under favorable conditions (Sener et al., 2002; Wraight & Clayton, 1974). Natural photosynthesis can guide the development of synthetic biology for renewable fuel production, but only if we can determine the structure-function relationships required for efficient solar-to-fuel energy conversion and build new structures that exploit this knowledge. Chl special pairs have attracted great interest as primary electron donors, but the complexity of natural photosystems makes it difficult to study these Chls directly. Model protein systems such as the water-soluble Chl protein (WSCP) and the B820 dimer of LH1 allow the investigation of excitonic interactions between Chls or between bacteriochlorophylls (BChls) without spectral congestion from other pigments (Bednarczyk et al., 2016; Chang et al., 1990; Ferretti et al., 2014; Pieper et al., 2011; Srivastava et al., 2021; Visschers et al., 1991), but these (B)Chl dimers are not structural mimics of special pairs. The challenges of studying special pairs have inspired chemists to synthesize numerous small molecule mimics (Boxer & Closs, 1976; Kobuke & Miyaji, 1994; McCleese et al., 2018; Sharma et al., 2021; Wasielewski et al., 1976), which have provided valuable insights, but these can be labor-intensive to synthesize, overlook the role of protein matrix effects which are important in native special pairs (Gorka et al., 2021), and lack the fine control over Chl-Chl distances and orientations needed to reproduce the precise geometries of native special pairs. *De novo*-designed Zn-tetrapyrrole monomer binding proteins (Ennist et al., 2017; Ennist, Stayrook., et al., 2022; Ennist, Zhao, et al., 2022; Farid et al., 2013; Fry et al., 2010; Kodali et al., 2017; Moser et al., 2016; Pirro et al., 2020; Polizzi et al., 2017) and *de novo* Chl dimer proteins (Cohen-Ofri et al., 2011; Curti et al., 2023; Rabanal et al., 1996; Wahadoszamen et al., 2014) have contributed to the understanding of light harvesting and quenching of excitation energy, but no structures of Chl dimers in designed proteins have been determined experimentally. Systematic methods of assembling Chl dimers with predefined geometries are lacking, making it difficult to correlate structure and function. Despite decades of active research, there has been no generalizable strategy to assemble Chl dimers that precisely match special pair geometries.

**Figure 1:**
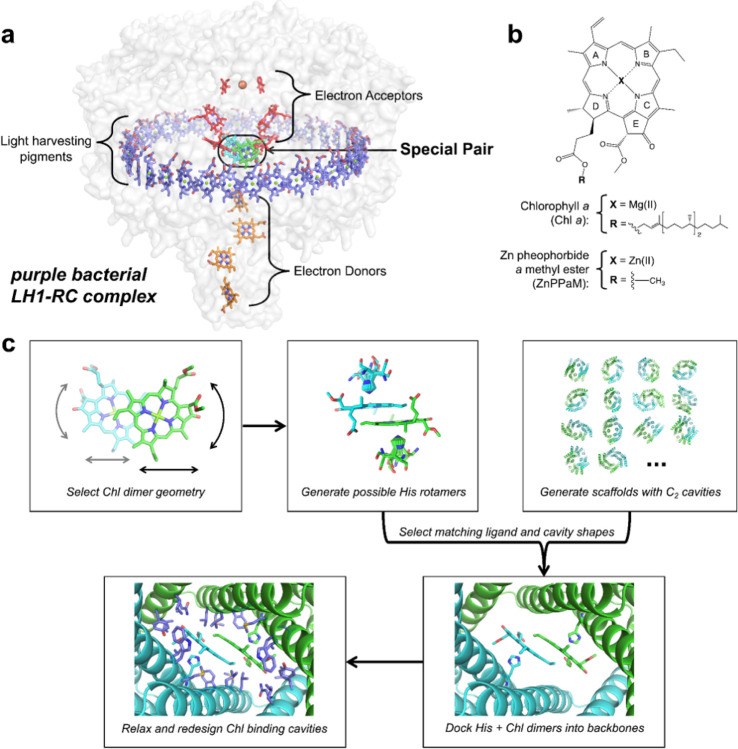
Computational design of Chl special pair proteins. (a) Cryo-electron microscopic (cryo-EM) structure of native photosynthetic LH1-RC complex from purple non-sulfur bacteria (*Blastochloris viridis*) (PDB ID: 6ET5) (Qian et al., 2018). The special pair is shown in green and cyan, electron acceptors in red, electron donors in orange, and light-harvesting pigments in blue. Hydrocarbon tail groups and carotenoids have been removed for clarity. (b) Chemical structures of two chlorin compounds, Chl *a* and Zn pheophorbide *a*methyl ester (ZnPPaM). Chl *a* and ZnPPaM have similar spectroscopic properties. (c) Computational design of Chl special pair proteins begins with selection of a Chl dimer geometry and generation of inverse His rotamers. His-Chl dimers are docked into designed homodimers, and the Chl binding pockets are redesigned using Rosetta FastDesign.

We reasoned that recent advances in computational protein design could enable the creation of stable, water-soluble proteins that assemble Chl dimers with predefined geometries and which can be built into extensive protein assemblies. Binding a small molecule as a dimer is a computational challenge, because the binding interface involves not just the protein but also the second small molecule, which has an independent set of rotational and translational degrees of freedom. To control these degrees of freedom, we sought to design homodimers with perfect two-fold cyclic (C_2_) symmetry, which bind a C_2_-symmetric Chl pair such that the C_2_ symmetry axes of the protein and chromophore are coincident, similar to native reaction centers, which can have true C_2_ symmetry (Chen et al., 2020; Gisriel et al., 2017) or pseudo-C_2_ symmetry (Figure 1a). C_2_ symmetry ensures that the two bound Chl molecules will have near-degenerate site energies, improving the resonance between pigment transitions necessary to create delocalized states (Reppert, 2023). For Chl dimer protein scaffolds, we chose hyperstable C_2_-symmetric repeat protein dimers containing symmetric pockets with tunable sizes and geometries (Brunette et al., 2015, 2020; Doyle et al., 2015; Fallas et al., 2017; Hicks et al., 2022). In this dimeric repeat protein architecture (Figure 1c), the hydrophobic core is independent from the small molecule binding site, enabling full customization for binding with little impact on the overall protein structure. Several thousand C_2_-symmetric homodimers that sample a wide range of superhelical curvature, rise, and radius parameters have been generated (Fallas et al., 2017; Hicks et al., 2022).

To probe the effect of geometry on Chl-Chl coupling, we set out to design a range of C_2_-symmetric dimers that hold two closely interacting Chl molecules in varied geometries including the arrangement found in native special pairs. In native proteins, (B)Chls typically have a pentacoordinate central Mg(II) or Zn(II) ion with a histidine (His) N_ε_ atom as the axial ligand. For each chosen special pair geometry, we built a His rotamer interaction field and stored the possible His-Chl interaction geometries in a hash table (Figure 1c; see Methods for details). For each geometrically compatible C_2_ scaffold, we cycled through His-Chl rotamers from the hash table, aligned them to the scaffold C_2_-symmetry axis, and searched for matches of the His N-C_α_-C backbone atoms to the backbone atoms of the residues lining the binding cavity. Scaffolds for which the His N-C_α_-C backbone atoms aligned with corresponding atoms in the protein backbone, and which could accommodate the Chl dimer without clashes, were redesigned using symmetric Rosetta FastDesign to optimize hydrophobic packing and hydrogen bonding around the Chls (DiMaio et al., 2011; Fleishman et al., 2011; Leaver-Fay et al., 2011; Leman et al., 2020; Maguire et al., 2021) (Figure 1c). Designs were filtered based on the Rosetta full-atom energy, the solvent-accessible surface area of the Chl dimer (DSasa), His rotamers, and His N_ε_-metal ligation geometry. We selected 43 designs based upon 13 unique scaffolds for experimental characterization (see Supplementary Table 1 for amino acid sequences). We also characterized an additional 5 variants generated based on structural information, as described below for SP3. The protein monomer sizes range from 20.6 to 28.4 kDa (179 to 261 amino acids). We refer to these 48 designs as Chl Special Pair proteins, or SP for brevity.

**Figure 2:**
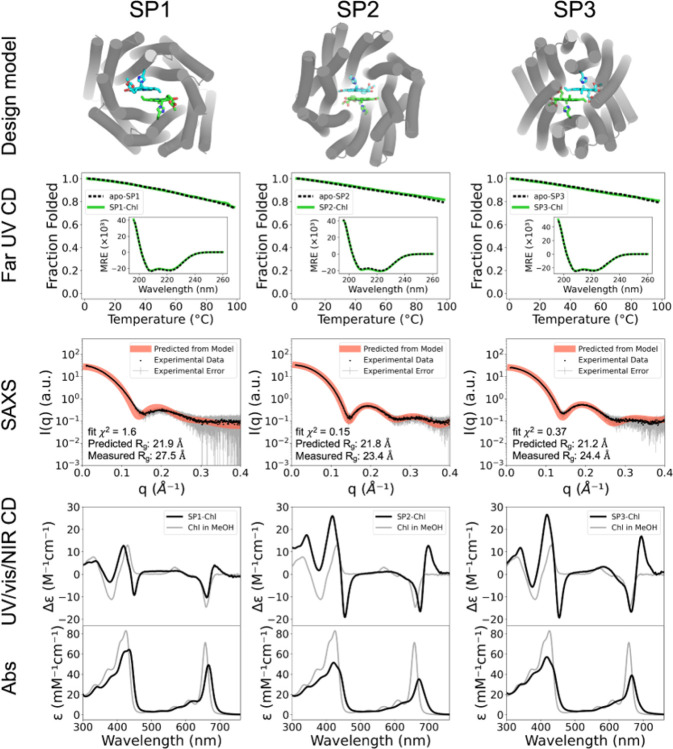
Folding, stability and ZnPPaM binding of SP proteins. SP protein design models (top) are displayed with α-helices represented as cylinders and Chl molecules as sticks. Far UV CD signals at 222 nm monitored with increasing temperature show proteins to be highly thermostable in both apo-states (black dashed traces) and ZnPPaM-bound states (green traces). Far UV CD spectra measured at 25°C (inset) have features typical of highly α-helical proteins including minima at 208 and 222 nm. Binding of ZnPPaM has little effect on secondary structure composition (green traces). Molar residue ellipticity (MRE) on the y-axis is given in deg cm^2^ dmol^−1^ residue^−1^. Experimental Small Angle X-ray Scattering (SAXS) data, shown as black points with error bars in gray, are in good agreement with SAXS profiles predicted from apo-state design models using the FoXS server, shown as red traces (Schneidman-Duhovny et al., 2013, 2016). The UV/vis/NIR CD spectrum of each protein in the ZnPPaM-bound state is shown in comparison to a control spectrum of ZnPPaM in methanol (MeOH) with the absorbance (Abs) spectrum of the same sample beneath. In each case, the protein-bound dimer acquires a positive band at ~690 nm, consistent with calculations based on dimer geometries (*vide infra*). Unbound ZnPPaM was removed by sterile filtration and PD-10 column chromatography prior to data collection.

Following SP protein expression in *E. coli*, SDS-PAGE gels showed that all 48 designs were present in the soluble fractions of lysates. Proteins were purified by Ni-NTA and size-exclusion chromatography (SEC) (Supplementary Figure 1). All SEC traces exhibited protein absorption at the elution volume expected for homodimer formation. Of 20 designs investigated by Small Angle X-ray Scattering (SAXS) in the apo-state, 15 had SAXS profiles suggesting a 3-dimensional shape consistent with the design model (Figure 2, Supplementary Figure 2, and Supplementary Table 2) (Schneidman-Duhovny et al., 2013, 2016). A slightly lower predicted radius of gyration (R_g_) value compared to experimental SAXS data is likely due to a dense hydration shell around the highly charged SP proteins (Kim et al., 2016; Svergun et al., 1998). The far ultraviolet (UV) circular dichroism (CD) spectra of three SP proteins that expressed in high yield (≥140 mg/L) show the proteins are highly α-helical with and without the synthetic Chl *a* derivative, Zn pheophorbide *a* methyl ester (ZnPPaM). Thermal denaturation curves monitored by the CD signal at 222 nm indicate that all three proteins are highly thermostable in the apo- and holo-states (Figure 2).

At longer wavelengths in the UV/visible/near-infrared (UV/vis/NIR), CD spectra can serve as a convenient probe of excitonic interactions between Chls. Monomeric Chls including Chl *a* and ZnPPaM exhibit asymmetric negative CD signals in the Q_y_ region near ~670 nm (Supplementary Figure 3) (Lindorfer et al., 2017). When Chl dimers are arranged in chiral protein environments, however, excitonic interactions can produce delocalized transitions with chiral character, yielding CD signals that are stronger and conservative (i.e., composed of a bisignate doublet that integrates to zero). Figure 2 shows that ZnPPaM bound to the SP1, SP2, and SP3 proteins have bisignate CD features in the Q_y_ region (in the red part of spectrum), consistent with excitonic coupling between the Chls. As shown in Supplementary Table 3, the Q_y_ CD features of SP2 and SP3 are substantially stronger relative to their Q_y_ absorption bands than is the Q_y_ CD signal of monomeric ZnPPaM in organic solvent. ZnPPaM binding titrations of SP2 and SP3 monitored by CD in the Q_y_ region show that the CD doublets are attributable to the binding of ZnPPaM dimers. Curve fitting of the CD titrations yields SP2-ZnPPaM dissociation constants (K_D_s) of 300 nM for K_D1_ and 2.5 μM for K_D2_, and SP3-ZnPPaM K_D_s of 800 nM for K_D1_ and 1.0 μM for K_D2_ (Supplementary Figure 4). Substantial changes in the absorbance spectrum of ZnPPaM on binding to SP2 made it possible to fit an absorbance titration, yielding K_D_s of 110 nM and 2.0 μM for K_D1_ and K_D2_, respectively, in agreement with the CD titration results (Supplementary Figure 5). Fluorescence titrations provided K_D_ estimates using a 1:1 binding model of protein monomer to ZnPPaM, giving an SP1-ZnPPaM K_D_ of 660 nM and an SP2-ZnPPaM K_D_ of 480 nM (Supplementary Figure 6).

**Figure 3:**
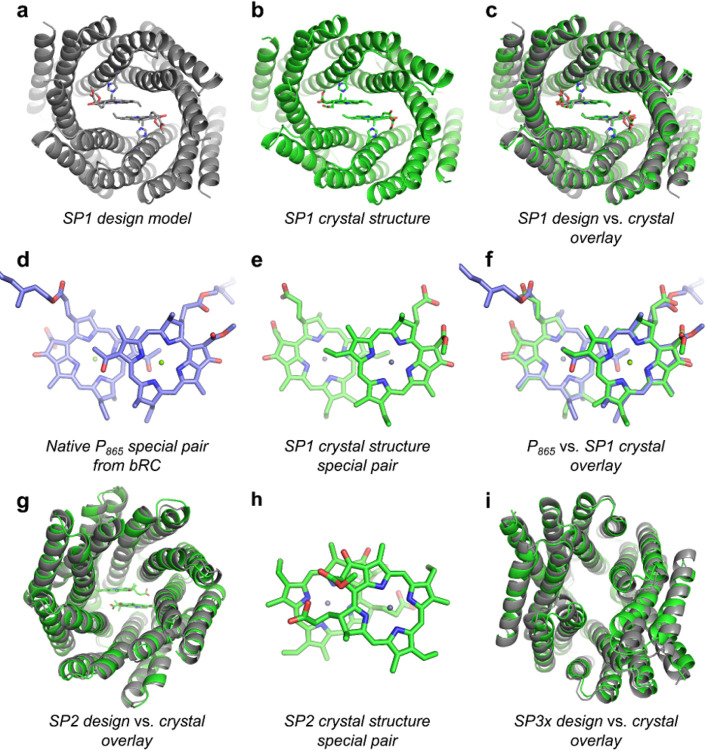
X-ray crystal structures of designed SP proteins. (a) Rosetta design model of SP1. (b) SP1 crystal structure at 2.0 Å resolution with ZnPPaM molecules bound (PDB ID: 7UNJ). (c) SP1 Rosetta design model (gray) aligns to SP1 crystal structure (green) with 1.6 Å C_α_ atom RMSD. (d) The BChl *a* special pair from a 2.5 Å resolution cryo-EM structure of the purple bacterial RC-LH1 complex (*Rhodobacter sphaeroides*) (PDB ID: 7PIL) (Qian et al., 2021). (e) The ZnPPaM dimer from the SP1 crystal structure shown in panel (b). (f) The ZnPPaM dimer of the SP1 crystal structure (green) aligns with the native purple bacterial special pair (blue) to 0.23 Å RMSD across corresponding atoms of the tetrapyrrole rings. (g) Rosetta design model of SP2 (gray) aligns to the holo-state SP2 crystal structure (green, PDB ID: 7UNI) with 1.4 Å C_α_ atom RMSD. (h) The ZnPPaM dimer in the SP2 crystal structure (green) deviates from the predicted dimer geometry (not shown; 3.5 Å RMSD). (i) Rosetta design model of SP3x (gray) aligns to apo-state SP3x crystal structure (green, PDB ID: 8EVM) with 1.6 Å C_α_ atom RMSD.

Based on the results of SEC, SAXS, and spectroscopic experiments (Figure 2 and Supplementary Figures 1–6), we selected promising candidates for X-ray crystallographic structure determination. We solved the crystal structures of 3 designs, SP1, SP2, and SP3x (a close relative of SP3), and found that all three had protein backbone conformations that matched the corresponding design models to within 1.7 Å C_α_ RMSD (Figure 3).

The X-ray crystal structure of SP1 was solved in the ZnPPaM-bound state to 2.0 Å resolution, revealing a special pair geometry closely matching that of purple photosynthetic bacteria (Figure 3a–f). The rotameric state of the Zn-ligating His121 is identical to that in the design model, and several hydrophobic and T-stacking interactions form as designed. Hydrogen bonds to the ring E ketone group, shown to be important for modulating special pair redox potentials(Lin et al., 1994), form with Gln10 in both ZnPPaM molecules. Alignment of the tetrapyrrole rings of the SP1-ZnPPaM dimer to nine native BChl *a* special pairs from different species of purple bacteria gave RMSDs of 0.23–0.28 Å (Cao et al., 2022; Niwa et al., 2014; Qian et al., 2021, 2022; Selikhanov et al., 2020; Swainsbury et al., 2021; Tani et al., 2020; Yu et al., 2018). (See details of RMSD measurements in Methods). For comparison, the special pairs of two crystal structures of the same *Thermochromatium tepidum* LH1-RC complex deviate from one another by 0.22 Å RMSD across the tetrapyrrole rings (PDB IDs: 3WMM and 5Y5S) (Niwa et al., 2014; Yu et al., 2018). The RMSD between the ZnPPaM dimer in the SP1 crystal structure and its design model is 0.25 Å.

SP2 was intended to assemble a ZnPPaM dimer with a conformation significantly different from native special pairs in order to investigate the effect of dimer geometry. The SP2 crystal structure was solved in both the apo-state and the ZnPPaM-bound state to 2.4 and 2.5 Å resolution, respectively. The apo- and holo-state amino acid backbones both agree with the SP2 design model to within 1.4 Å RMSD (Figure 3g). The holo-state crystal structure has two copies of the SP2 dimer in the asymmetric unit; alignment of the two ZnPPaM dimers shows their binding geometries are equivalent, with an RMSD of 0.22 Å over the tetrapyrrole rings. The ZnPPaM molecules are ligated by His178 as in the SP2 design model. After alignment of the crystal structure and design model protein backbones, the corresponding tetrapyrrole rings are approximately coplanar. Despite the accuracy of the protein backbone design, the crystal structure shows the ZnPPaM molecules are rotated and translated relative to the design model (Figure 3h). Compared to the apo-state crystal structure, the SP2 binding cavity widens by ~1.6 Å in the presence of ZnPPaM; this expansion provides the extra volume needed for the ZnPPaM molecules to adopt their unexpected conformation. While the ZnPPaM dimer in SP2 differs from the design model, the crystal structure nevertheless satisfies the objective of creating a non-native dimer geometry.

An apo-state crystal structure of SP3x, which shares 94% sequence identity with SP3, was solved to 3.05 Å resolution. The SP3x homodimeric design model agrees with the crystal structure to 1.61 Å C_α_ RMSD (Figure 3i). The crystallized SP3x protein fails to bind a ZnPPaM dimer; CD studies indicate that it can only bind one ZnPPaM molecule per protein dimer. While the design model largely matches the crystal structure, subtle discrepancies in the shape of the binding pocket and side chain rotameric states may contribute to sub-stoichiometric ZnPPaM binding. With the high-resolution SP3x structure in hand, we redesigned the binding cleft, allowing Rosetta FastRelax to select new binding site residues and make small adjustments to the dimer geometry. We tested a new set of five mutants, one of which, SP3, exhibits high affinity assembly of a ZnPPaM dimer, as assayed by CD titration (Supplementary Figure 4). SP3 is designed to assemble a Chl dimer similar in structure to the P700 special pair of Photosystem I. Alignment of the SP3 design model to the P700 special pair from the crystal structure of Photosystem I (PDB ID: 1JB0) (Jordan et al., 2001) gives an RMSD of 0.68 Å across tetrapyrrole ring atoms. We grew green holo-state crystals of SP3 with ZnPPaM bound but were unable to solve the structure.

**Figure 4:**
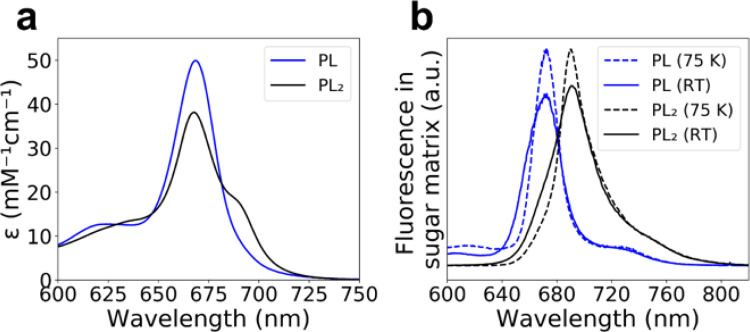
Spectral shift on ZnPPaM dimer binding in SP2 protein. (a) Molar absorptivity in solution of ZnPPaM when bound to SP2 as a monomer (PL; blue trace) with 0.3 equivalents of ZnPPaM present per SP2 dimer and as a ZnPPaM dimer (PL_2_; black trace) with 2.0 equivalents of ZnPPaM present per SP2 dimer. Monomer and dimer traces are fitted spectra from a binding titration experiment with 150 mM NaCl and 10 mM Tris buffer at pH 8 (see Supplementary Figure 5). (b) Temperature dependence of fluorescence emission spectra of SP2 suspended in a sucrose/trehalose matrix with ZnPPaM monomer (PL; blue) and dimer (PL_2_; black) with the same protein-to-ZnPPaM ratios as in panel (a). Samples were photoexcited at 405 nm. Fluorescence emission intensities are scaled so that the emission maxima at 75 K are equal for the monomer and dimer samples. (See Methods and Supplementary Figure 7 for details).

The absorption and fluorescence spectra of native special pairs are shifted compared to monomeric (B)Chls, in part due to excitonic coupling between the (B)Chls, which enables them to act as exciton traps (Gorka et al., 2021; Taylor & Kassal, 2019; van Amerongen et al., 2000). The SP2-ZnPPaM dimer absorbance spectrum presents a red-shifted shoulder in solution. Analysis of SP2-ZnPPaM absorbance binding titrations (Figure 4a and Supplementary Figure 5) shows that whereas the Q_y_ transition of monomeric ZnPPaM in SP2 has an absorbance maximum at 669 nm with an extinction coefficient (ε_669nm_) of 49,900 M^−1^cm^−1^, the SP2-ZnPPaM dimer spectrum has its Q_y_ maximum slightly shifted to 668 nm with a decreased ε_668nm_ of 38,200 M^−1^cm^−1^. While the monomer has no discernable spectroscopic feature at 690 nm (its ε_690nm_ is 9,400 M^−1^cm^−1^), the SP2-ZnPPaM dimer spectrum has a distinct shoulder with ε_690nm_ of 17,700 M^−1^cm^−1^.

To rule out the possibility that the SP2-ZnPPaM bands at 668 and 690 nm may represent two populations of distinct ZnPPaM oligomers, we investigated the SP2-ZnPPaM spectral features using low-temperature spectroscopy in a sucrose/trehalose matrix (Figure 4b and Supplementary Figure 7; see Methods for details). If the two Q_y_ bands originate from the same ZnPPaM dimer species, decreasing temperature should increase the fluorescence emission intensity at the lower energy transition relative to the higher energy transition due to relaxation of the electronic excitation within the dimer. We prepared a dimer sample with 2.0 molar equivalents of ZnPPaM per SP2 protein dimer and a monomer sample with only 0.3 molar equivalents per protein dimer, both in sucrose/trehalose films. The monomer sample lacked a red-shifted shoulder, and its Q_y_ fluorescence maximum at 672 nm intensified with decreasing temperature from room temperature (RT) to 75 K (Figure 4b and Supplementary Figure 7). This behavior is expected for monomeric chromophores, because lower temperatures decrease homogeneous line broadening. In contrast, in the SP2-ZnPPaM dimer sample, two emission bands were observed at 673 and 692 nm, and lowering the temperature decreased the intensity of dimer fluorescence emission at 673 nm while increasing emission at 692 nm. In effect, the temperature dependence of the fluorescence intensity near 672 nm is reversed in the dimer compared to the monomer (Figure 4b and Supplementary Figure 7j). This indicates that the two spectroscopic features in the SP2-ZnPPaM dimer are coupled; lower temperatures disfavor thermal promotion from the lower energy 692 nm state to the higher energy state, causing 692 nm fluorescence emission to intensify while 673 nm emission weakens.

We calculated theoretical CD spectra based on the ZnPPaM dimer geometries of design models and crystal structures and compared them to experimental CD traces. We found that the signs of the CD Cotton effects predicted from the crystal structures of SP1 and SP2 are consistent with the experimental signs in the Q_y_ region. In the SP3 dimer, the calculated spectrum based on the design model agrees with the experimentally-measured signs of the CD Cotton effects, suggesting that the P700-like ZnPPaM dimer in SP3 assembles as designed. (See Methods and Supplementary Figures 8–10 for details of CD spectral calculations). The excitonic coupling strengths of the SP1- and SP2-ZnPPaM dimer crystal structures were calculated to be 87 and 241 cm^−1^, respectively. The experimental absorbance spectrum of SP2-ZnPPaM has features at 668 and 690 nm, corresponding to a Q_y_ peak splitting of 477 cm^−1^, or a coupling of 239 cm^−1^, consistent with the calculated coupling strength. In addition, calculations based on the SP2-ZnPPaM crystal structure predict an oscillator strength redistribution towards the high-energy excitonic component (Supplementary Figure 11). This results in a band centered at ~690 nm with a lower intensity than the high-energy component at ~668 nm, in good agreement with the observed absorbance spectrum in which the 690 nm band appears as a weaker shoulder (Figure 4a).

**Figure 5:**
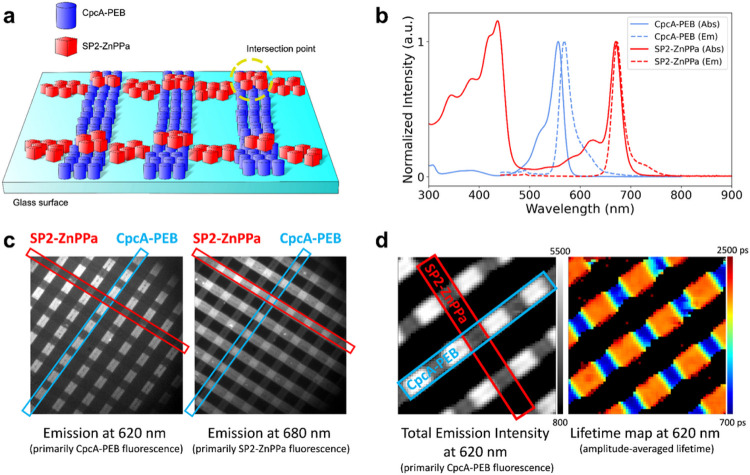
SP2 functions as an energy transfer acceptor for native light harvesting protein. (a) Schematic of contact-printed proteins on functionalized glass surface depicts lines of CpcA-PEB with separate rows of SP2-ZnPPa printed on top. (b) Normalized absorbance (Abs) and fluorescence emission (Em) spectra of CpcA-PEB and SP2-ZnPPa. Spectra were collected in solution prior to contact printing on glass surface. (c) Fluorescence intensities of printed cross-pattern of SP2-ZnPPa and CpcA-PEB at 620 nm and 680 nm with excitation by a 450 nm wide-field LED. (d) Fluorescence intensity and lifetime map monitored at 620 nm with excitation by 485 nm laser. Scale bars represent absolute fluorescence intensity in a.u. (left) and fluorescence lifetime in ps (right).

Native special pairs play a critical role as energy transfer acceptors for antenna proteins. To test whether our designed SP proteins participate in energy transfer with natural light-harvesting proteins, we analyzed excitation energy transfer in 2D surface arrays using Fluorescence Lifetime Imaging Microscopy (FLIM) in combination with nanoimprint lithography (Huang et al., 2020) (Figure 5). We selected the cyanobacterial antenna protein CpcA with attached phycoerythrobilin (PEB) as the energy transfer donor. CpcA-PEB was purified from *E. coli*, and gives a strong fluorescence emission maximum at 568 nm and emission extending past 630 nm (Barnett et al., 2017), overlapping with the excitation spectrum of Zn pheophorbide *a* (ZnPPa) when bound to SP2. Notably, Q_y_ peak splitting was not observed in SP2 assembled with ZnPPa instead of ZnPPaM (Figure 5b), suggesting that the peripheral substituents of the chlorin play a significant role in excitonic coupling. To monitor energy transfer, ~5 μm-wide linear arrays of CpcA-PEB and perpendicular linear arrays of SP2-ZnPPa were applied to a poly-L-lysine functionalized glass surface by contact printing (Huang et al., 2020), creating intersection points where CpcA-PEB and SP2-ZnPPa interact and other locations in which only one of the proteins was present. Wide-field epifluorescence imaging with excitation at 450 nm was used to analyze the surface attachment; filtering emission at 620 nm preferentially displays regions of CpcA-PEB, while 680 nm preferentially shows SP2-ZnPPa. At the intersections between the lines, donor CpcA-PEB emission (620 nm filter) was decreased and SP2-ZnPPa acceptor (680 nm filter) was increased, indicating energy transfer from donor to acceptor (Figure 5c).

To quantify the strength of the interaction between CpcA-PEB and SP2-ZnPPa, we used time-resolved single photon counting. The surface was illuminated with a 485 nm picosecond laser filtered at 620 nm (donor emission), and photons were counted for individual pixels (surface resolution approximately 300 nm) over time, allowing both total fluorescence intensity and lifetime to be analyzed (Figure 5d). In regions with only CpcA-PEB, fluorescence intensity was above 4000 arbitrary units (a.u.) and the lifetime was over 2 ns (τ_av_ = 2058 ps). Regions with both CpcA-PEB and SP2-ZnPPa show reduced fluorescence intensity (<1000 a.u.) and lifetimes under 0.9 ns (τ_av_ = 839 ps). We estimate the energy transfer efficiency of CpcA-PEB to SP2-ZnPPa in 2D arrays to be 59%, similar to figures seen for natural fluorescent proteins (Becker et al., 2004; Duncan et al., 2004; Tramier et al., 2002) (see Supplementary Figure 12 for time-resolved photon counting and energy transfer calculation).

For efficient solar energy conversion, nature organizes photosynthetic machinery into specialized compartments such as thylakoids in plants or chromatophore vesicles in purple photosynthetic bacteria (Singharoy et al., 2019). As a first step towards such structures, we sought to incorporate a Chl-binding SP protein into a two-component supercomplex with octahedral symmetry (King et al, 2014). The C_2_ symmetry axes of 12 copies of the SP2 dimer and the C_3_ axes of 8 copies of a C_3_-symmetric homotrimer (Boyken et al., 2016, 2019) were aligned with the C_2_ and C_3_ axes of an octahedron. We sampled rotations and translations along these axes to generate a closely packed octahedral model with the C_2_ dimers on the edges and the C_3_ trimers on the vertices. Interface residues were redesigned to create binding surfaces between the SP2 dimers and the trimers. Twenty-one designs were experimentally characterized, and one was found to assemble into octahedral structures by negative-stain EM (Supplementary Figures 13–14). In this nanocage, the SP2-like component shares 87% sequence identity with the original SP2 design, and its absorbance spectrum has a red-shifted shoulder in the Q_y_ region, similar to the original SP2-ZnPPaM complex (Supplementary Figure 13).

**Figure 6:**
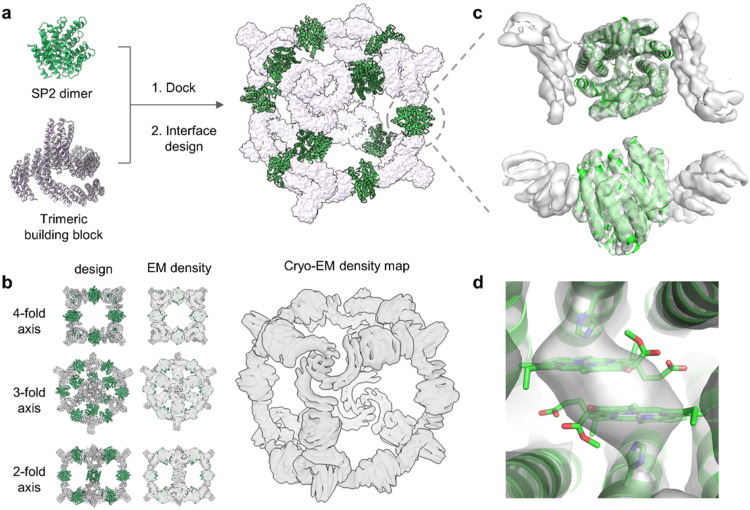
Cryo-EM structure of a nanocage that assembles Chl dimer proteins. (a) Nanocage design model was generated by docking SP2 dimer with trimeric building block to form 2-component octahedral nanocage architecture. Rosetta sequence design was used to stabilize interfaces between dimeric and trimeric building blocks. (b) Multiple views of the nanocage design model (left), design model docked into the cryo-EM density (middle), and cryo-EM density alone (right) (EMDB ID: EMD-40208, Map #1). The cryo-EM map (gray) closely matches the design model (color). (c) Axial and lateral views of the C_2_ dimer unit. The holo-state crystal structure of SP2 (green, PDB ID: 7UNI) was fitted into the EM density map of the nanocage (gray surface; EMDB ID: EMD-40209, Map #2). (d) Close-up view of the Chl binding pocket indicates that the ZnPPaM dimer structure in the nanocage EM density map (gray surface; Map #2) is consistent with the SP2 crystal structure (green).

The cryo-EM structure of the 600 kDa octahedral nanocage with 24 ZnPPaM bound was solved to intermediate resolution (average resolution 6.5 Å) in which helices are well resolved. The design model accurately predicts protein-protein interfaces and the overall architecture (Figure 6a,b and Supplementary Figures 15–16). The asymmetric unit of the cryo-EM structure agrees with the design model to 3.4 Å backbone RMSD. Variability analysis showed several modes of flexibility which may have limited the resolution (see Supplementary Information for movies of protein breathing motions). While the resolution is not sufficient to confidently determine the orientations of the Chls, the cryo-EM density map is consistent with the Chl dimer geometry in the SP2 crystal structure (Figure 6c,d).

## Conclusion

We describe the first designed proteins which hold Chl dimers in precisely defined closely juxtaposed geometries. We obtain crystal structures of two holo-state designs: the first, SP1, reproduces the binding geometry of the native purple bacterial reaction center special pair with sub-angstrom precision, and the second, SP2, has a distinct geometry with the Chls closer together. Our use of symmetry reduces the complexity of the design procedure while ensuring equivalent site energies for the two bound Chls to strengthen excitonic coupling. Symmetry also enables integration of the *de novo* special pair proteins into larger supercomplexes. Our octahedral nanocage incorporating 12 ZnPPaM dimers is a first step towards *de novo* design of photosynthetic compartments analogous to thylakoids or chromatophores.

SP2 exhibits spectroscopic hallmarks of native special pairs including Cotton effects by CD, shifting of absorption and fluorescence bands, and energy transfer activity when paired with the native antenna protein CpcA. SP1 exhibits weaker excitonic coupling than SP2 and the BChl special pair of purple photosynthetic bacteria despite its close structural similarity to the latter. The stronger coupling of SP2 relative to SP1 may reflect the closer spacing of the ZnPPaM molecules in SP2, whereas the stronger coupling of the purple bacterial special pair is due to the stronger Q_y_ transition dipole moment of bacteriochlorins as compared to chlorins (Knox & Spring, 2003). Prediction of the spectroscopic properties of a Chl dimer in a protein is complicated by the fact that Chl-Chl coupling energies are typically similar in magnitude to the available thermal energy, Frank-Condon active vibrational and phonon reorganization energies, and local Chl vibrational frequencies (Reppert, 2023). Accurate optical predictions require benchmarking of theoretical methods using robust model systems. Native photosynthetic proteins can be difficult to isolate and typically contain many interacting pigment molecules, creating spectral congestion. The highly thermostable, water-soluble Chl dimer proteins described herein avoid the complexity of native pigment-protein complexes and provide a testbed to investigate structure-spectrum relationships.

Studies of photosynthetic light harvesting and charge separation indicate that natural photosynthesis leaves room for efficiency improvements (Barber & Tran, 2013; Blankenship et al., 2011; Ennist, Stayrook., et al., 2022; Hitchcock et al., 2022; Romero et al., 2017). The successful design of excitonically coupled chromophore pairs, and the assembly of these into organized superstructures, suggests that *de novo* protein design could provide a route to new solar-to-fuel energy conversion technologies. With its red-shifted absorbance spectrum, SP2 is well tuned to accept energy from light-harvesting Chls or other pigments (Figure 5), and it has the long-lived excited state (>3 ns fluorescence emission lifetime; Supplementary Figure 7) needed to allow electron transfer to occur. To couple light absorption to charge separation for solar fuel production, the next step is to engineer transfer of the excited state electron to a low potential electron acceptor.

## Methods

### Computational placement of the chlorophyll special pair into symmetric protein scaffolds:

Identifying residue positions capable of accommodating the Chl special pair which is scalable to millions of potential scaffolds was achieved by utilizing a motif-hash based method (Fallas et al., 2017; Yao et al., 2022) specifically adopted for the histidine-Chl dimer motif inspired by the special pair of purple bacteria, P865. However, the number of example structures of the histidine-Chl dimer motif found in the PDB is not acceptable for effectively populating a motif hash table. Therefore, generating additional structural examples of the symmetric histidine-Chl dimer complex was generated *de novo*.

The conformer generation was achieved using the NeRF algorithm (available on github at https://github.com/atom-moyer/nerf) which translates internal molecular coordinates to global molecular coordinates. Various conformers were generated by varying the internal coordinates such as the relative positioning of the Chl groups, the dihedral of ligation by the histidine residue, and the rotamer of the histidine sidechain. The full complex was duplicated along the C_2_ axis to create the symmetric complex. If the relative orientations of the Chls were varied, clashes between the rings and their substitutions were evaluated and filtered. The full process of *de novo* motif generation was repeated for ligation with the epsilon and delta nitrogen of the imidazole ring.

Once the *de novo* conformers were generated, the 6-D transformation that defines the relative orientation of the N-CA-C atoms of the ligating histidine residues were hashed using a method described previously (Fallas et al., 2017; Yao et al., 2022). The hashed 6-D transformation was used as a key in a multi value hash table (https://github.com/atom-moyer/getpy), and the associated value was a vector that defined the information necessary to rebuild the histidine-Chl complex, which nitrogen from the histidine was used for ligation and the internal coordinates of the histidine rotamer.

During evaluation of design scaffolds, the 6-D transformation of each symmetric residue pair across chains was evaluated and hashed using the same method used to hash the *de novo* conformers described above. That allowed the identification of symmetric residue pairs which have similar 6-D transformations to the potentially acceptable ligation geometries. If a matching 6-D transformation was found, the histidine-Chl complex was rebuilt from the associated value in the hash table, and the complex was evaluated in the context of the protein. If the Chls did not clash with the backbone atoms of the protein, the placement was accepted and passed into the protein design process.

A python package and example scripts which generate the *de novo* hash tables and place the histidine-Chl complexes into symmetric proteins can be found here: https://github.com/atom-moyer/stapler.

### Protein expression and purification:

Synthetic genes with N-terminal His_6_ tags followed by TEV protease cleavage sites were purchased in pET29b expression vectors from Integrated DNA Technologies, Inc. Plasmids were transformed into Lemo21(DE3) Competent *E. coli* (New England Biolabs). For each protein, a single *E. coli* colony was grown in a culture of 5 mL of LB with 100 μg/mL kanamycin overnight at 37°C. Overnight cultures were used to inoculate 50–500 mL cultures of auto-induction media (Studier, 2005). Bacteria were grown in auto-induction media at 37°C with shaking for 4 hours, then incubated shaking overnight at 18°C. Bacteria were harvested and resuspended in 300 mM NaCl, 30 mM imidazole, 25 mM Tris buffer at pH 8, ~0.01 mg/mL DNase (Sigma-Aldrich), ~0.1 mg/mL lysozyme (Sigma-Aldrich), and Pierce^™^ Protease Inhibitor Tablets (Thermo Fisher Scientific). Bacteria were lysed by sonication and centrifuged at ~18,000 g for 30 minutes. Soluble fractions were purified by Immobilized Metal Affinity Chromatography (IMAC) gravity columns packed with Ni-NTA agarose resin (Qiagen) at room temperature. Columns were washed with a buffer containing 20 mM imidazole and proteins were eluted with a 300 mM imidazole buffer. Samples were digested with His-tagged TEV protease in the presence of 0.5 mM dithiothreitol for 1–2 days at room temperature. Digested proteins were buffer exchanged into 20 mM imidazole buffer, 300 mM NaCl, and 25 mM Tris buffer at pH 8 and applied to IMAC columns to remove TEV protease and uncleaved protein. Proteins were further purified by size-exclusion chromatography (SEC) using an ÄKTA FPLC with a Superdex 200 Increase 10/300 GL column (GE Healthcare Life Sciences). Protein and Chl molecular weights were verified by reverse-phase liquid chromatography/mass spectrometry (LC/MS) with an Agilent G6230B TOF instrument using an AdvanceBio RP-Desalting column. Mass spectra were deconvoluted in Bioconfirm using a total entropy algorithm (Supplementary Figures 17–19).

### Protein-chlorophyll sample preparation:

Zn pheophorbide a methyl ester (ZnPPaM) was purchased from Frontier Scientific, Inc. ZnPPaM stock solutions were prepared in dimethyl sulfoxide (DMSO) or methanol to concentrations between 200 μM and 1 mM. ZnPPaM concentrations were determined using mass measurements and by using the known absorptivity of Zn pheophytin *a*, which has a similar absorbance spectrum and an extinction coefficient ε_659nm_ of 77,300 M^−1^cm^−1^ in 80% acetone/20% deionized water (Jones et al., 1976). Ultraviolet/visible (UV/vis) absorbance spectra were collected using a Jasco V-750 spectrophotometer with a 1 nm bandwidth and 400 nm/min scanning speed. Protein-ZnPPaM complexes were prepared by slowly adding freshly-prepared ZnPPaM stock solution to protein solution in aqueous buffer at room temperature and incubating samples for several hours. Unbound ZnPPaM was removed by centrifugation to pellet precipitated ZnPPaM, sterile filtration using a 0.22 μm syringe filter, and/or running a PD-10 desalting column purification (Sephadex^™^ G-25 M resin, Cytiva Life Sciences).

### Circular Dichroism (CD) spectroscopy:

CD spectra were collected using a Jasco J-1500 spectrophotometer. For protein secondary structure assays, spectra were measured on samples of 0.2–0.4 mg/mL protein in 1 mm quartz cuvettes from 260 to 190 nm with a 1 nm bandwidth, 1 nm data interval, data integration time (DIT) of 1 second, and scanning speed of 50 nm/min. Thermal melts were monitored at 222 nm from 2 to 98°C with a 2 nm bandwidth and 8 second DIT. UV/vis/near-infrared (NIR) CD transitions of protein-bound Chls were examined in the 800–300 nm region in 1 cm quartz cuvettes as averages of 10 scans using a 3 nm bandwidth, 1 nm data interval, DIT of 4 seconds, and scanning speed of 50 nm/min unless otherwise noted. UV/vis/NIR CD spectra shown in Figure 2 were collected after sterile filtering with 0.22 μm filter and PD-10 desalting column purification (Sephadex^™^ G-25 M resin, Cytiva Life Sciences). Each spectrum represents the average of two independent sample preparations. Samples contained 8–15 μM protein (monomer concentration) with equimolar ZnPPaM, 150 mM NaCl, and 10 mM Tris buffer at pH 8. ZnPPaM dry powder was dissolved in methanol stock solutions immediately before adding to protein solutions. Samples were allowed to incubate for 8–16 hours at room temperature prior to measuring spectra.

### Small angle X-ray scattering (SAXS):

Data were collected at the Advanced Light Source (ALS) using the SIBYLS beamline for high throughput SAXS (Dyer et al., 2014). Proteins were sent as 30 μL samples in 96-well plates with buffer-matched blank solutions for background subtraction. Data sets were processed in SAXS Frameslice version 1.4.13 and compared to design models using FoXS (Schneidman-Duhovny et al., 2013, 2016).

### Fluorescence quantum yield measurements:

Fluorescence spectra displayed in Supplementary Figure 6 were recorded on a Fluorolog Horiba Jobin Yvon spectrofluorimeter equipped with a Xenon lamp, a double monochromator and a photomultiplier detector. The experiments were carried out in right angle (RA) configuration. Each baseline subtracted fluorescence spectrum was corrected for spectral sensitivity of the fluorimeter and re-absorption by assuming the middle of the cuvette is the origin of emission. Relative quantum yields were estimated using Chl *a* in diethyl ether as a reference (Weber & Teale, 1957).

### Low-temperature absorbance and fluorescence spectroscopy in a sucrose/trehalose film:

Solutions of SP2-ZnPPaM were mixed with a saturated sugar solution made by dissolving a 50:50 sucrose/trehalose (w/w) in distilled water as described previously (Caram et al., 2016). A 100 μL sample of SP2 at 34 mg/mL (ZnPPaM dimer) or 156 mg/mL (ZnPPaM monomer) was added dropwise to 100 μL of the sugar solution and gently mixed. The sugar/protein mixture was dropped onto a 0.1 mm quartz cuvette from Starna Cells Inc. and kept under vacuum in the dark for 24 hr. The sample was then loaded into a Janis ST-100 cryostat using a custom-built copper cuvette holder and cooled with liquid nitrogen. A Lakeshore 330 Autotuning Temperature Controller was used to control the temperature. An Agilent Cary-60 spectrometer was used to collect all absorbance spectra across temperatures. For the temperature-dependent fluorescence emission spectra, a home-built setup equipped with a Thorlabs 405 nm laser head (CPS405). The collected emission was fiber coupled into a Flame Ocean Optics spectrometer. Lifetimes were recorded using a homebuilt, all-reflective epifluorescence setup. The samples were excited via a pulsed laser output from a 405 nm pulsed diode laser (LDH-P-C-405, PicoQuant) with a 10 MHz repetition rate. The emission was subsequently filtered with a 420 nm longpass dichroic beam splitter (DMLP425R, Thorlabs) and 420 nm longpass filter (10CGA-420, Newport). The emission was detected by avalanche photodiodes (PD050-CTD, Micro Photon Devices). Time-correlated single photon counting (TCSPC) traces were histogrammed using a Picoquant HydraHarp 400 and analyzed via the corresponding software.

### Molecular dynamics simulations:

All molecular dynamics (MD) simulations were performed with Amber18 (Case et al., 2018) using the ff14SB forcefield (Maier et al., 2015) for proteins and TIP3P (Jorgensen et al., 1983) for water. To obtain the forcefield parameters of the chromophore (ZnPPaM), we used the MCPB.py module of Amber (Li & Merz, 2016). Atomic charges were calculated using the restrained electrostatic potential (RESP) fitting scheme, while force constants were calculated with the Seminario method. QM geometry optimization and ESP calculations were performed with Gaussian 16 (Rev B.01) (Frisch et al., 2016) at the B3LYP/6–31G* level. Parameters for the organic part of the chromophore were taken from the general AMBER force field (GAFF).

As starting structures, either design models or crystal structures were employed. Protonation states were determined with the H++ webserver, at pH 8 (using default parameters) (Anandakrishnan et al., 2012; Gordon et al., 2005; Myers et al., 2006). Topology and geometry files were generated with LEaP, using an isometric truncated-octahedron shape for the periodic box, with a minimum distance between the protein and the edges of the box of 1.5 nm. Protein charges were neutralized with Na^+^ and Cl^−^ ions.

Minimization and initial equilibration steps were performed following a recently developed protocol (Roe & Brooks, 2020). Briefly, it consists of nine sequential energy minimizations and short MD runs, which sum 4000 steps of minimization and 40000 MD steps (totalling 30 ps), followed by a final MD equilibration (500000 steps, 1000 ps). Then, after discarding the first 200 ns, production runs were done in the NPT ensemble at 300.0 K, with a time step of 2 fs, and constraining bonds involving hydrogen atoms via the SHAKE algorithm. Constant temperature and pressure were ensured with the Langevin thermostat (collision frequency: 2 ps^−1^) and Monte Carlo barostat, respectively. Long-range electrostatics were considered via the Particle Mesh Ewald (PME) model, setting the direct space sum cut-off to 1.0 nm.

### Calculation of Chl dimer excitonic coupling and spectra:

Calculations involving excited states were performed on the chromophore geometries of the design models and on the crystal structures. In the latter case, hydrogen atoms were added using UCSF Chimera 1.11 (Pettersen et al., 2004). Electronic couplings were calculated using the EET (electronic energy transfer) module from Gaussian, at the CAM-B3LYP/6–31G* level. The effect of the environment was considered through the polarizable continuum model (PCM) (Iozzi et al., 2004; Tomasi et al., 2005), choosing n-octanol as representative of the protein dielectric behavior. The EET analysis considered six singlet excited states per chromophore.

To obtain circular dichroism spectra we used the results of the EET calculations and the Excitonic Analysis Tool (EXAT) program (Jurinovich et al., 2014, 2015, 2018). Rotatory strengths were calculated by considering both electric and magnetic dipoles in the velocity formulation. Spectral lineshapes were simulated as gaussians, with a full-width at half-maximum of 350 cm^−1^ for the Q_y_ and Q_x_ transitions and 1150 cm^−1^ for transitions in the Soret region. Spectra were shifted by −0.25 eV to reproduce the experimental position of the Q_y_ band.

### Fluorescence lifetime imaging (FLIM):

Fluorescence lifetime imaging was conducted on a home-built laser scanning time-resolved fluorescence microscope as described previously (Huang et al., 2020). The microscope was equipped with a 485 nm picosecond diode laser (PicoQuant, PDL 828) and a 450 nm LED (Thorlabs, M470L2) (wide-field illumination) as excitation sources. The excitation light was focused by a 100 × objective (PlaneFluorite, NA = 1.4, oil immersion, Olympus). The emitted light was filtered using a 495 nm dichroic beam-splitter (Semrock) and 565/25, 630/20 and 680/45 nm bandpass filters (Semrock) to remove the background excitation light. The microscope was fitted with a spectrometer (150 lines/mm grating, Acton SP2558, Princeton Instruments) and an electron-multiplying charge-coupled device (EMCCD) camera (ProEM 512, Princeton Instruments) for emission spectrum acquisition and wide-field imaging. A hybrid detector (HPM-100–50, Becker & Hickl) was used for single-photon counting. The modulation of the excitation laser was synchronized with a time-correlated single-photon counting (TCSPC) module (SPC-150, Becker & Hickl) for the lifetime decay measurement. The repetition rate of the laser was set at 1 MHz. The excitation laser power was adjusted to produce a fluence of approximately 2×10^14^ photons pulse^−1^ cm^−2^. The instrument response function (IRF) of the set up was approximately 130 ps. Fluorescence lifetime images were recorded by scanning the excitation laser over the sample using a piezo scanner. The FLIM data was analyzed using OriginPro (OriginLab Corporation) and FLIMfit (www.flimfit.org).

### X-ray crystallography for SP1 and SP2:

Crystals of SP1 and SP2 were grown using protein purified as described above. Protein samples dispensed in 1 μL drops at purification concentrations were mixed with equal volume of a crystallization solution and set in hanging drops (refer to Supplementary Table 4 for conditions). Vapor phase equilibration of the resulting drops against a 1 mL reservoir of the same crystallization solution resulted in growth of crystals. The crystals were flash cooled in liquid nitrogen. Diffraction data were collected on a Pilatus area detector at the Advanced Light Source (ALS) synchrotron facility at beamline 5.0.2 for SP1-ZnPPaM and SP2-ZnPPaM protein assemblies. Diffraction data were collected on a Rigaku HyPix-6000HE hybrid photon counting detector at the Fred Hutchinson Cancer Center (Fred Hutch) for SP2. The resulting data sets (Supplementary Table 4) extend to 2.0 Å, 2.4 Å, and 2.5 Å resolution for SP1-ZnPPaM, apo-state SP2, and SP2-ZnPPaM, respectively. The asymmetric units of the SP1-ZnPPaM and apo-state SP2 structures each contained one complete dimer (two copies of a protein subunit), and the SP2-ZnPPaM structure had 2 dimers in the asymmetric unit.

Data were processed using HKL2000 (Otwinowski & Minor, 1997) or Aimless (Evans & Murshudov, 2013). The placement of subunits was determined using the molecular replacement algorithm in program PHENIX (Adams et al., 2010). Local rebuilding of all constructs was performed using the program COOT (Emsley et al., 2010), followed by refinement in PHENIX (Adams et al., 2010). For the ZnPPaM-bound structures, the protein was built and refined completely with waters (excluding waters from the binding site) and other chemicals before manually fitting ZnPPaM into the density that remained. ZnPPaM energies were calculated using eLBOW (Moriarty et al., 2009). The final values for R_work_ / R_free_ are notated in Supplementary Table 4.

### X-ray crystallography for SP3x:

All crystallization experiments for the SP3x protein were conducted using the sitting drop vapor diffusion method. Crystallization trials were set up in 200 nL drops using the 96-well plate format at 20°C. Crystallization plates were set up using a Mosquito from SPT Labtech, then imaged using UVEX microscopes and UVEX PS-600 from JAN Scientific. Diffraction quality SP3x crystals formed in 2.4 M sodium malonate dibasic monohydrate pH 7.0.

Diffraction data were collected at the Advanced Light Source at beamline 5.0.1. X-ray intensities and data reduction were evaluated and integrated using XDS (Kabsch, 2010) and merged/scaled using Pointless/Aimless in the CCP4 program suite (Winn et al., 2011). Structure determination and refinement starting phases were obtained by molecular replacement using Phaser (McCoy et al., 2007) using the designed model for the structures. Following molecular replacement, the models were improved using phenix.autobuild (Adams et al., 2010); efforts were made to reduce model bias by setting rebuild-in-place to false, and using simulated annealing and prime-and-switch phasing. Structures were refined in Phenix (Adams et al., 2010). Model building was performed using COOT (Emsley & Cowtan, 2004). The final model was evaluated using MolProbity (Williams et al., 2018). Data collection and refinement statistics are recorded in Supplementary Table 4. Data deposition, atomic coordinates, and structure factors reported for the SP3x protein in this paper have been deposited in the Protein Data Bank (PDB), http://www.rcsb.org/ with accession code 8EVM.

### Protein structure alignment:

Protein crystal structures were compared to Rosetta design models by aligning corresponding backbone C_α_ atoms and calculating RMSDs using TM-align (Zhang & Skolnick, 2005). (B)Chl special pair geometries were compared using the align function in The PyMOL Molecular Graphics System, Version 2.5.2, Schrödinger, LLC. To facilitate comparison of the geometries of special pairs composed of different (B)Chl types, omit unimportant conformational differences such as rotameric states of peripheral substituents, and neglect differences in the Mg(II) *vs*. Zn(II) positions, only the atoms of the tetrapyrrole rings were considered in pairwise special pair structural alignments. These atoms included the 4 pyrrole nitrogen atoms, 16 pyrrole carbon atoms, and 4 methine bridge carbons from each (B)Chl monomer, giving 48 atoms per (B)Chl dimer that were used for structural comparisons.

Corresponding atoms were aligned in PyMOL and the RMSD over all 48 atom pairs was calculated. Native BChl *a* special pairs used for comparison to the SP1 protein came from 5 different species of purple photosynthetic bacteria, including *Rhodobacter sphaeroides*, *Rhodopseudomonas palustris*, *Thermochromatium tepidum*, *Gemmatimonas phototrophica*, and *Thiorhodovibrio* strain 970. The PDB IDs of the nine X-ray crystal and cryo-EM structures containing the native special pairs used for comparison to SP1 were: 7PIL, 7VNY, 6Z27, 6Z02, 6Z5S, 3WMM, 5Y5S, 7O0U, and 7C9R (Cao et al., 2022; Niwa et al., 2014; Qian et al., 2021, 2022; Selikhanov et al., 2020; Swainsbury et al., 2021; Tani et al., 2020; Yu et al., 2018).

### Nanocage design:

The Chl binding dimer SP2 was docked against a library of trimeric cyclic oligomer scaffolds (C_3_) from previous *de novo* designs (Boyken et al., 2016; Fallas et al., 2017; Hsia et al., 2021) to form octahedral cages (O_32_) using the RPXDock software (Sheffl er et al., 2022). The RPXdock package utilizes a hierarchical sampling strategy to search for interfaces with high shape complementarity based on residue pair transform scoring. The top 10 scored docking configurations for each scaffold were subsequently sequence designed by symmetric RosettaDesign calculations, using a previously reported protocol (King et al., 2014) to carry out two-component protein-protein interface design. Briefly, we aim to design low-energy, well-packed hydrophobic protein-protein interfaces where protein building blocks are treated as rigid backbones and only side chain rotamers of interface residues are packed with layer design restrictions. The beta_nov16 or a clash-fixed score function was used during the design. Finally, all cage designs were filtered based on shape complementarity (>0.6), interface surface area (solvent-accessible surface area, 1000 < sasa <1600), predicted binding energy (ddG <−20 kcal/mol), buried unsatisfied hydrogen bonds (uhb <3), and clash check (< 3). All Rosetta scripts used are available upon request.

### Transmission negative-stain electron microscopy (nsEM) and image processing:

SEC purified cage fractions were diluted to about 0.5 μM (monomeric component concentration) for negative-stain EM characterization. Briefly, on a glow-discharged formvar/carbon supported 400-mesh copper grid (Ted Pella, Inc.), 6 μL of protein sample were drop-casted for 2 mins. The grid was blotted and stained with 3 μL of 2% uranyl formate, blotted again, and stained with 3 μL of uranyl formate for 20 s before final blotting. Micrographs of stained samples were taken on a 120kV Talos L120C transmission electron microscope. All nsEM datasets were collected using the EPU software and processed by CryoSparc (Punjani et al., 2017) with contrast transfer function (CTF) correction. All the particle picks were 2D classified for 20 iterations into 50 classes. Particles from selected classes were used for building the ab-initio initial model. The initial model was homogeneously refined using C_1_ and the corresponding O symmetry.

### Cryo-EM grid preparation and data collection:

Grids (QUANTIFOIL^®^ R 2/2 on Cu 300 mesh grids + 2 nm C) were vitrified using a Vitrobot Mark IV with chamber maintained at 22°C and 100% humidity. Grids were plunge-frozen into liquid ethane directly following application of 3.5 μl of the ZnPPaM-loaded nanocage to the glow-discharged (for 5 s) surface of the grid. Grids were screened at the NYU Cryo-EM core facility using a Talos Arctica microscope operated at 200 kV with a Gatan K3 camera. Data were then collected on a Titan Krios microscope operated at 300 kV with a Gatan K3 camera with BioQuantum imaging filter (“Krios 2” at the New York Structural Biology Center). Data were acquired from duplicate grids using Leginon (Suloway et al., 2005) and pre-processed (2X binned and motion-corrected with MotionCor2 (Zheng et al., 2017) within Appion (Lander et al., 2009). Full data collection parameters are shown in Supplementary Table 5.

### Cryo-EM data processing and model building:

Aligned and dose-weighted micrographs were imported to Cryosparc v.3 (Punjani et al., 2017) and processed using the workflow shown in Supplementary Figure 15. During data collection, we noted a high proportion of damaged (compressed or fragmented) nanocage particles in areas of ice with a reported ALS thickness below 40 nm. Curation of micrographs to exclude those with the thinnest ice, and with CTF fit resolution lower than ~6Å, facilitated picking of intact nanocage particles. 2D classification was performed on manually-picked particles to generate templates representing diverse views of the nanocage, but subsequent template-based picking tended to exclude rare particle views. Recovery of these rare views was improved by using a single template for picking representing the view most often missed in prior template-based picking efforts (see Supplementary Table 6). Compared to picking with multiple templates, using a single, rare-view template improved the recovery of diverse particle views, which were then used as a training set for Topaz (Bepler et al., 2019). Picking with Topaz yielded diverse, well-centered nanocage particles. Data from each of the two grids imaged were picked with Topaz separately, and the curated particles were then combined and further curated in 2D. This larger set of curated particles was used to retrain Topaz (204,039 vs. 19,355 in initial Topaz training set) on the full set of micrographs from both grids. Particles picked using this Topaz model were then curated by 2D classification, micrograph curation by ice thickness and CTF fit values, and removal of duplicates.

A 200-micrograph subset from a single grid was used to generate an *ab initio* 3D reconstruction. Following iterative rounds of homogeneous and heterogeneous refinement, this map served as the initial 3D model for processing of the full particle set from both grids. 3D refinement and classification yielded a map of the full nanocage with an average reported resolution of ~6.5Å (as calculated in Cryosparc using gold-standard FSC cutoff of 0.143). O symmetry was imposed during the final round of refinement. Continuous conformational heterogeneity likely limited the resolution of the full nanocage map, as discrete states were not readily separable by further 3D classification. Multiple modes of flexibility were visualized using Cryosparc’s 3D Variability Analysis (Punjani & Fleet, 2021), supporting the notion that the nanocage particles used in refinement were subject to compression/deformation (see Supplementary Information for movies of protein breathing motions). We then used partial signal subtraction and focused refinement to improve resolution in the ligand-binding region of the cage (region enclosed in yellow mask, Supplementary Figure 15 inset). Prior to partial signal subtraction, particles were expanded with T symmetry (the highest-order symmetry containing a complete Chl-binding dimer). The symmetry-expanded, partially-subtracted particle set was then refined in C_1_ using Local Refinement in Cryosparc.

The cryo-EM map of the full nanocage was used for real-space refinement of a model in Phenix (Echols et al., 2012). Due to the intermediate map resolution, all residues were modeled as alanine and restraints (secondary structure, Ramachandran, and non-crystallographic symmetry constraints) were imposed during refinement. The designed nanocage model was used as a starting point for refinement, and individual chains were docked into cryo-EM maps using Chimera (Pettersen et al., 2004) before hydrogen removal and truncation to polyalanine using phenix.pdbtools. Stubbed, docked models were then subjected to restrained real-space refinement in Phenix. We observed a notable difference between the design model and the cryoEM density in the angle between each trimeric interface helix and its attached DHR “arm”. To generate a starting model for restrained refinement of the full nanocage, we first performed rigid-body refinement, with each trimer subunit modeled as two rigid bodies (corresponding to the interface helix and “arm” regions; residues 259–337 and 1–258, respectively). Cryo-EM model statistics are listed in Supplementary Table 7.

## Data Availability

X-ray crystallographic coordinates and data files of designed SP dimer proteins were deposited at the Protein Data Bank (PDB) with accession codes 7UNJ (SP1 with ZnPPaM bound), 7UNH (SP2, apo-state), 7UNI (SP2 with ZnPPaM bound), and 8EVM (SP3x, apo-state). An electron microscopy map of the full ZnPPaM-binding nanocage (Map #1) was deposited in the Electron Microscopy Data Bank with accession code EMD-40208, and a backbone model was deposited in the Protein Data Bank with accession code 8GLT. An electron microscopy map of the ZnPPaM-binding region of the nanocage (Map #2) was deposited in the Electron Microscopy Data Bank with accession code EMD-40209.

## References

[1] Adams P. D., Afonine P. V., Bunkóczi G., Chen V. B., Davis I. W., Echols N., Headd J. J., Hung L.-W., Kapral G. J., Grosse-Kunstleve R. W., McCoy A. J., Moriarty N. W., Oeffner R., Read R. J., Richardson D. C., Richardson J. S., Terwilliger T. C., & Zwart P. H. (2010). PHENIX: a comprehensive Python-based system for macromolecular structure solution. Acta Crystallographica. Section D, Biological Crystallography, 66(Pt 2), 213–221. 20124702 10.1107/S0907444909052925 PMC2815670

[2] Álvarez-Moreno M., de Graaf C., López N., Maseras F., Poblet J. M., & Bo C. (2015). Managing the computational chemistry big data problem: the ioChem-BD platform. Journal of Chemical Information and Modeling, 55(1), 95–103. 25469626 10.1021/ci500593j

[3] Anandakrishnan R., Aguilar B., & Onufriev A. V. (2012). H++ 3.0: automating pK prediction and the preparation of biomolecular structures for atomistic molecular modeling and simulations. Nucleic Acids Research, 40(Web Server issue), W537–W541. 22570416 10.1093/nar/gks375 PMC3394296

[4] Barber J., & Tran P. D. (2013). From natural to artificial photosynthesis. Journal of the Royal Society, Interface / the Royal Society, 10(81), 20120984. 10.1098/rsif.2012.0984 PMC3627107 23365193

[5] Barnett S. F. H., Hitchcock A., Mandal A. K., Vasilev C., Yuen J. M., Morby J., Brindley A. A., Niedzwiedzki D. M., Bryant D. A., Cadby A. J., Holten D., & Hunter C. N. (2017). Repurposing a photosynthetic antenna protein as a super-resolution microscopy label. Scientific Reports, 7(1), 16807. 29196704 10.1038/s41598-017-16834-z PMC5711914

[6] Becker W., Bergmann A., Hink M. A., König K., Benndorf K., & Biskup C. (2004). Fluorescence lifetime imaging by time-correlated single-photon counting. Microscopy Research and Technique, 63(1), 58–66. 14677134 10.1002/jemt.10421

[7] Bednarczyk D., Dym O., Prabahar V., Peleg Y., Pike D. H., & Noy D. (2016). Fine tuning of chlorophyll spectra by protein-induced ring deformation. Angewandte Chemie, 55(24), 6901–6905. 27098554 10.1002/anie.201512001 PMC6690836

[8] Bepler T., Morin A., Rapp M., Brasch J., Shapiro L., Noble A. J., & Berger B. (2019). Positive-unlabeled convolutional neural networks for particle picking in cryo-electron micrographs. Nature Methods, 16(11), 1153–1160. 31591578 10.1038/s41592-019-0575-8 PMC6858545

[9] Blankenship R. E., Tiede D. M., Barber J., Brudvig G. W., Fleming G., Ghirardi M., Gunner M. R., Junge W., Kramer D. M., Melis A., Moore T. A., Moser C. C., Nocera D. G., Nozik A. J., Ort D. R., Parson W. W., Prince R. C., & Sayre R. T. (2011). Comparing photosynthetic and photovoltaic efficiencies and recognizing the potential for improvement. Science, 332(6031), 805–809. 21566184 10.1126/science.1200165

[10] Boxer S. G., & Closs G. L. (1976). A covalently bound dimeric derivative of pyrochlorophyllide a. A Possible model for reaction center chlorophyll. Journal of the American Chemical Society, 98(17), 5406–5408.

[11] Boyken S. E., Benhaim M. A., Busch F., Jia M., Bick M. J., Choi H., Klima J. C., Chen Z., Walkey C., Mileant A., Sahasrabuddhe A., Wei K. Y., Hodge E. A., Byron S., Quijano-Rubio A., Sankaran B., King N. P., Lippincott-Schwartz J., Wysocki V. H., … Baker D. (2019). De novo design of tunable, pH-driven conformational changes. Science, 364(6441), 658–664. 31097662 10.1126/science.aav7897 PMC7072037

[12] Boyken S. E., Chen Z., Groves B., Langan R. A., Oberdorfer G., Ford A., Gilmore J. M., Xu C., DiMaio F., Pereira J. H., Sankaran B., Seelig G., Zwart P. H., & Baker D. (2016). De novo design of protein homo-oligomers with modular hydrogen-bond network-mediated specificity. Science, 352(6286), 680–687. 27151862 10.1126/science.aad8865 PMC5497568

[13] Brunette T. J., Bick M. J., Hansen J. M., Chow C. M., Kollman J. M., & Baker D. (2020). Modular repeat protein sculpting using rigid helical junctions. Proceedings of the National Academy of Sciences of the United States of America, 117(16), 8870–8875. 32245816 10.1073/pnas.1908768117 PMC7183188

[14] Brunette T. J., Parmeggiani F., Huang P.-S., Bhabha G., Ekiert D. C., Tsutakawa S. E., Hura G. L., Tainer J. A., & Baker D. (2015). Exploring the repeat protein universe through computational protein design. Nature, 528(7583), 580–584. 26675729 10.1038/nature16162 PMC4845728

[15] Cao P., Bracun L., Yamagata A., Christianson B. M., Negami T., Zou B., Terada T., Canniffe D. P., Shirouzu M., Li M., & Liu L.-N. (2022). Structural basis for the assembly and quinone transport mechanisms of the dimeric photosynthetic RC–LH1 supercomplex. Nature Communications, 13(1), 1–12. 10.1038/s41467-022-29563-3 PMC9007983 35418573

[16] Caram J. R., Doria S., Eisele D. M., Freyria F. S., Sinclair T. S., Rebentrost P., Lloyd S., & Bawendi M. G. (2016). Room-Temperature Micron-Scale Exciton Migration in a Stabilized Emissive Molecular Aggregate. Nano Letters, 16(11), 6808–6815. 27689389 10.1021/acs.nanolett.6b02529

[17] Case D. A., Ben-Shalom I. Y., Brozell S. R., Cerutti D. S., Cheatham T. E. III, Cruzeiro V. W. D., Darden T. A., Duke R. E., Ghoreishi D., Gilson M. K., Gohlke H., Goetz A. W., Greene D., Harris R., Homeyer N., Huang Y., Izadi S., Kovalenko A., … Kollman P. A. (2018). AMBER 2018. University of California, San Francisco.

[18] Chang M. C., Callahan P. M., Parkes-Loach P. S., Cotton T. M., & Loach P. A. (1990). Spectroscopic characterization of the light-harvesting complex of Rhodospirillum rubrum and its structural subunit. Biochemistry, 29(2), 421–429. 2105744 10.1021/bi00454a017

[19] Chen J.-H., Wu H., Xu C., Liu X.-C., Huang Z., Chang S., Wang W., Han G., Kuang T., Shen J.-R., & Zhang X. (2020). Architecture of the photosynthetic complex from a green sulfur bacterium. Science, 370(6519), eabb6350. 33214250 10.1126/science.abb6350

[20] Cohen-Ofri I., van Gastel M., Grzyb J., Brandis A., Pinkas I., Lubitz W., & Noy D. (2011). Zinc-bacteriochlorophyllide dimers in de novo designed four-helix bundle proteins. A model system for natural light energy harvesting and dissipation. Journal of the American Chemical Society, 133(24), 9526–9535. 21563814 10.1021/ja202054m

[21] Croce R., & van Amerongen H. (2014). Natural strategies for photosynthetic light harvesting. Nature Chemical Biology, 10(7), 492–501. 24937067 10.1038/nchembio.1555

[22] Curti M., Maffeis V., Duarte L. G. T. A., Shareef S., Hallado L. X., Curutchet C., & Romero E. (2023). Engineering Excitonically-Coupled Dimers in an Artificial Protein for Light Harvesting via Computational Modelling. Protein Science: A Publication of the Protein Society, e4579. 36715022 10.1002/pro.4579 PMC9951196

[23] DiMaio F., Leaver-Fay A., Bradley P., Baker D., & André I. (2011). Modeling symmetric macromolecular structures in Rosetta3. PloS One, 6(6), e20450. 21731614 10.1371/journal.pone.0020450 PMC3120754

[24] Doyle L., Hallinan J., Bolduc J., Parmeggiani F., Baker D., Stoddard B. L., & Bradley P. (2015). Rational design of α-helical tandem repeat proteins with closed architectures. Nature, 528(7583), 585–588. 26675735 10.1038/nature16191 PMC4727831

[25] Duncan R. R., Bergmann A., Cousin M. A., Apps D. K., & Shipston M. J. (2004). Multi-dimensional time-correlated single photon counting (TCSPC) fluorescence lifetime imaging microscopy (FLIM) to detect FRET in cells. Journal of Microscopy, 215(Pt 1), 1–12. 15230870 10.1111/j.0022-2720.2004.01343.x PMC1903372

[26] Dyer K. N., Hammel M., Rambo R. P., Tsutakawa S. E., Rodic I., Classen S., Tainer J. A., & Hura G. L. (2014). High-throughput SAXS for the characterization of biomolecules in solution: a practical approach. Methods in Molecular Biology, 1091, 245–258. 24203338 10.1007/978-1-62703-691-7_18 PMC4057279

[27] Echols N., Grosse-Kunstleve R. W., Afonine P. V., Bunkóczi G., Chen V. B., Headd J. J., McCoy A. J., Moriarty N. W., Read R. J., Richardson D. C., Richardson J. S., Terwilliger T. C., & Adams P. D. (2012). Graphical tools for macromolecular crystallography in PHENIX. Journal of Applied Crystallography, 45(Pt 3), 581–586. 22675231 10.1107/S0021889812017293 PMC3359726

[28] Emsley P., & Cowtan K. (2004). Coot: model-building tools for molecular graphics. Acta Crystallographica. Section D, Biological Crystallography, 60(Pt 12 Pt 1), 2126–2132. 15572765 10.1107/S0907444904019158

[29] Emsley P., Lohkamp B., Scott W. G., & Cowtan K. (2010). Features and development of Coot. Acta Crystallographica. Section D, Biological Crystallography, 66(Pt 4), 486–501. 20383002 10.1107/S0907444910007493 PMC2852313

[30] Ennist N. M., Mancini J. A., Auman D. B., Bialas C., Iwanicki M. J., Esipova T. V., Discher B. M., Moser C. C., & Dutton P. L. (2017). Maquette Strategy for Creation of Light- and Redox-Active Proteins. In Photosynthesis and Bioenergetics (pp. 1–33). WORLD SCIENTIFIC.

[31] Ennist N. M., Stayrook S. E., Dutton P. L., & Moser C. C. (2022). Rational design of photosynthetic reaction center protein maquettes. Frontiers in Molecular Biosciences, 9. 10.3389/fmolb.2022.997295 PMC9532970 36213121

[32] Ennist N. M., Zhao Z., Stayrook S. E., Discher B. M., Leslie Dutton P., & Moser C. C. (2022). De novo protein design of photochemical reaction centers. In Nature Communications (Vol. 13, Issue 1). 10.1038/s41467-022-32710-5 PMC9399245 35999239

[33] Evans P. R., & Murshudov G. N. (2013). How good are my data and what is the resolution? Acta Crystallographica. Section D, Biological Crystallography, 69(Pt 7), 1204–1214. 23793146 10.1107/S0907444913000061 PMC3689523

[34] Fallas J. A., Ueda G., Sheffl er W., Nguyen V., McNamara D. E., Sankaran B., Pereira J. H., Parmeggiani F., Brunette T. J., Cascio D., Yeates T. R., Zwart P., & Baker D. (2017). Computational design of self-assembling cyclic protein homo-oligomers. Nature Chemistry, 9(4), 353–360. 10.1038/nchem.2673 PMC5367466 28338692

[35] Farid T. A., Kodali G., Solomon L. A., Lichtenstein B. R., Sheehan M. M., Fry B. A., Bialas C., Ennist N. M., Siedlecki J. A., Zhao Z., Stetz M. A., Valentine K. G., Anderson J. L. R., Wand A. J., Discher B. M., Moser C. C., & Dutton P. L. (2013). Elementary tetrahelical protein design for diverse oxidoreductase functions. Nature Chemical Biology, 9(12), 826–833. 24121554 10.1038/nchembio.1362 PMC4034760

[36] Ferretti M., Novoderezhkin V. I., Romero E., Augulis R., Pandit A., Zigmantas D., & van Grondelle R. (2014). The nature of coherences in the B820 bacteriochlorophyll dimer revealed by two-dimensional electronic spectroscopy. Physical Chemistry Chemical Physics: PCCP, 16(21), 9930–9939. 24430275 10.1039/c3cp54634a

[37] Fleishman S. J., Leaver-Fay A., Corn J. E., Strauch E.-M., Khare S. D., Koga N., Ashworth J., Murphy P., Richter F., Lemmon G., Meiler J., & Baker D. (2011). RosettaScripts: a scripting language interface to the Rosetta macromolecular modeling suite. PloS One, 6(6), e20161. 21731610 10.1371/journal.pone.0020161 PMC3123292

[38] Frisch M. J., Trucks G. W., Schlegel H. B., Scuseria G. E., Robb M. A., Cheeseman J. R., Scalmani G., Barone V., Petersson G. A., Nakatsuji H., Li X., Caricato M., Marenich A. V., Bloino J., Janesko B. G., Gomperts R., Mennucci B., Hratchian H. P., Ortiz J. V., … Fox D. J. (2016). Gaussian 16 (Version Revision B.01) [Computer software].

[39] Fry H. C., Lehmann A., Saven J. G., DeGrado W. F., & Therien M. J. (2010). Computational design and elaboration of a de novo heterotetrameric alpha-helical protein that selectively binds an emissive abiological (porphinato)zinc chromophore. Journal of the American Chemical Society, 132(11), 3997–4005. 20192195 10.1021/ja907407m PMC2856663

[40] Gisriel C., Sarrou I., Ferlez B., Golbeck J. H., Redding K. E., & Fromme R. (2017). Structure of a symmetric photosynthetic reaction center-photosystem. Science, 357(6355), 1021–1025. 28751471 10.1126/science.aan5611

[41] Gordon J. C., Myers J. B., Folta T., Shoja V., Heath L. S., & Onufriev A. (2005). H++: a server for estimating pKas and adding missing hydrogens to macromolecules. Nucleic Acids Research, 33(Web Server issue), W368–W371. 15980491 10.1093/nar/gki464 PMC1160225

[42] Gorka M., Baldansuren A., Malnati A., Gruszecki E., Golbeck J. H., & Lakshmi K. V. (2021). Shedding Light on Primary Donors in Photosynthetic Reaction Centers. Frontiers in Microbiology, 12, 735666. 34659164 10.3389/fmicb.2021.735666 PMC8517396

[43] Hicks D. R., Kennedy M. A., Thompson K. A., DeWitt M., Coventry B., Kang A., Bera A. K., Brunette T. J., Sankaran B., Stoddard B., & Baker D. (2022). De novo design of protein homodimers containing tunable symmetric protein pockets. Proceedings of the National Academy of Sciences, 119(30), e2113400119. 10.1073/pnas.2113400119 PMC9335249 35862457

[44] Hitchcock A., Hunter C. N., Sobotka R., Komenda J., Dann M., & Leister D. (2022). Redesigning the photosynthetic light reactions to enhance photosynthesis - the PhotoRedesign consortium. The Plant Journal: For Cell and Molecular Biology, 109(1), 23–34. 34709696 10.1111/tpj.15552

[45] Hsia Y., Mout R., Sheffl er W., Edman N. I., Vulovic I., Park Y.-J., Redler R. L., Bick M. J., Bera A. K., Courbet A., Kang A., Brunette T. J., Nattermann U., Tsai E., Saleem A., Chow C. M., Ekiert D., Bhabha G., Veesler D., & Baker D. (2021). Design of multi-scale protein complexes by hierarchical building block fusion. Nature Communications, 12(1), 2294. 10.1038/s41467-021-22276-z PMC8052403 33863889

[46] Huang X., Vasilev C., & Hunter C. N. (2020). Excitation energy transfer between monomolecular layers of light harvesting LH2 and LH1-reaction centre complexes printed on a glass substrate. Lab on a Chip, 20(14), 2529–2538. 32662473 10.1039/d0lc00156b

[47] Iozzi M. F., Mennucci B., Tomasi J., & Cammi R. (2004). Excitation energy transfer (EET) between molecules in condensed matter: A novel application of the polarizable continuum model (PCM). The Journal of Chemical Physics, 120(15), 7029–7040. 15267604 10.1063/1.1669389

[48] Jones I. D., White R. C., Gibbs E., & Butler L. S. (1976). Estimation of zinc pheophytins, chlorophylls, and pheophytins in mixtures in diethyl ether or 80% acetone by spectrophotometry and fluorometry. Journal of Agricultural and Food Chemistry, 25(1), 146–149. 1002914 10.1021/jf60209a025

[49] Jordan P., Fromme P., Witt H. T., Klukas O., Saenger W., & Krauss N. (2001). Three-dimensional structure of cyanobacterial photosystem I at 2.5 A resolution. Nature, 411(6840), 909–917. 11418848 10.1038/35082000

[50] Jorgensen W. L., Chandrasekhar J., Madura J. D., Impey R. W., & Klein M. L. (1983). Comparison of simple potential functions for simulating liquid water. The Journal of Chemical Physics, 79(2), 926–935.

[51] Jurinovich S., Cupellini L., Guido C. A., & Mennucci B. (2018). EXAT: EXcitonic analysis tool. Journal of Computational Chemistry, 39(5), 279–286. 29151259 10.1002/jcc.25118

[52] Jurinovich S., Guido C. A., Bruhn T., Pescitelli G., & Mennucci B. (2015). The role of magnetic–electric coupling in exciton-coupled ECD spectra: the case of bis-phenanthrenes. Chemical Communications, 51(52), 10498–10501. 26033039 10.1039/c5cc03167b

[53] Jurinovich S., Pescitelli G., Di Bari L., & Mennucci B. (2014). A TDDFT/MMPol/PCM model for the simulation of exciton-coupled circular dichroism spectra. Physical Chemistry Chemical Physics: PCCP, 16(31), 16407–16418. 24603889 10.1039/c3cp55428g

[54] Kabsch W. (2010). XDS. Acta Crystallographica. Section D, Biological Crystallography, 66(Pt 2), 125–132. 20124692 10.1107/S0907444909047337 PMC2815665

[55] Kim H. S., Martel A., Girard E., Moulin M., Härtlein M., Madern D., Blackledge M., Franzetti B., & Gabel F. (2016). SAXS/SANS on Supercharged Proteins Reveals Residue-Specific Modifications of the Hydration Shell. Biophysical Journal, 110(10), 2185–2194. 27224484 10.1016/j.bpj.2016.04.013 PMC4880798

[56] King N. P., Bale J. B., Sheffler W., McNamara D. E., Gonen S., Gonen T., Yeates T. O., & Baker D. (2014). Accurate design of co-assembling multi-component protein nanomaterials. Nature, 510(7503), 103–108. 24870237 10.1038/nature13404 PMC4137318

[57] Knox R. S., & Spring B. Q. (2003). Dipole strengths in the chlorophylls. Photochemistry and Photobiology, 77(5), 497–501. 12812291 10.1562/0031-8655(2003)077<0497:dsitc>2.0.co;2

[58] Kobuke Y., & Miyaji H. (1994). Supramolecular Organization of Imidazolyl-Porphyrin to a Slipped Cofacial Dimer. Journal of the American Chemical Society, 116(9), 4111–4112.

[59] Kodali G., Mancini J. A., Solomon L. A., Episova T. V., Roach N., Hobbs C. J., Wagner P., Mass O. A., Aravindu K., Barnsley J. E., Gordon K. C., Officer D. L., Dutton P. L., & Moser C. C. (2017). Design and engineering of water-soluble light-harvesting protein maquettes. Chemical Science, 8(1), 316–324. 28261441 10.1039/c6sc02417c PMC5330312

[60] Lander G. C., Stagg S. M., Voss N. R., Cheng A., Fellmann D., Pulokas J., Yoshioka C., Irving C., Mulder A., Lau P.-W., Lyumkis D., Potter C. S., & Carragher B. (2009). Appion: an integrated, database-driven pipeline to facilitate EM image processing. Journal of Structural Biology, 166(1), 95–102. 19263523 10.1016/j.jsb.2009.01.002 PMC2775544

[61] Leaver-Fay A., Tyka M., Lewis S. M., Lange O. F., Thompson J., Jacak R., Kaufman K., Renfrew P. D., Smith C. A., Sheffl er W., Davis I. W., Cooper S., Treuille A., Mandell D. J., Richter F., Ban Y.-E. A., Fleishman S. J., Corn J. E., Kim D. E., … Bradley P. (2011). ROSETTA3: an object-oriented software suite for the simulation and design of macromolecules. Methods in Enzymology, 487, 545–574. 21187238 10.1016/B978-0-12-381270-4.00019-6 PMC4083816

[62] Leman J. K., Weitzner B. D., Lewis S. M., Adolf-Bryfogle J., Alam N., Alford R. F., Aprahamian M., Baker D., Barlow K. A., Barth P., Basanta B., Bender B. J., Blacklock K., Bonet J., Boyken S. E., Bradley P., Bystroff C., Conway P., Cooper S., … Bonneau R. (2020). Macromolecular modeling and design in Rosetta: recent methods and frameworks. Nature Methods, 17(7), 665–680. 32483333 10.1038/s41592-020-0848-2 PMC7603796

[63] Lindorfer D., Müh F., & Renger T. (2017). Origin of non-conservative circular dichroism of the CP29 antenna complex of photosystem II. Physical Chemistry Chemical Physics: PCCP, 19(11), 7524–7536. 28247880 10.1039/c6cp08778g

[64] Lin X., Murchison H. A., Nagarajan V., Parson W. W., Allen J. P., & Williams J. C. (1994). Specific alteration of the oxidation potential of the electron donor in reaction centers from Rhodobacter sphaeroides. Proceedings of the National Academy of Sciences of the United States of America, 91(22), 10265–10269. 7937938 10.1073/pnas.91.22.10265 PMC45000

[65] Li P., & Merz K. M. Jr. (2016). MCPB.py: A Python Based Metal Center Parameter Builder. Journal of Chemical Information and Modeling, 56(4), 599–604. 26913476 10.1021/acs.jcim.5b00674

[66] Maguire J. B., Haddox H. K., Strickland D., Halabiya S. F., Coventry B., Griffin J. R., Pulavarti S. V. S. R. K., Cummins M., Thieker D. F., Klavins E., Szyperski T., DiMaio F., Baker D., & Kuhlman B. (2021). Perturbing the energy landscape for improved packing during computational protein design. Proteins, 89(4), 436–449. 33249652 10.1002/prot.26030 PMC8299543

[67] Maier J. A., Martinez C., Kasavajhala K., Wickstrom L., Hauser K. E., & Simmerling C. (2015). ff14SB: Improving the Accuracy of Protein Side Chain and Backbone Parameters from ff99SB. Journal of Chemical Theory and Computation, 11(8), 3696–3713. 26574453 10.1021/acs.jctc.5b00255 PMC4821407

[68] McCleese C., Yu Z., Esemoto N. N., Kolodziej C., Maiti B., Bhandari S., Dunietz B. D., Burda C., & Ptaszek M. (2018). Excitonic Interactions in Bacteriochlorin Homo-Dyads Enable Charge Transfer: A New Approach to the Artificial Photosynthetic Special Pair. The Journal of Physical Chemistry. B, 122(14), 4131–4140. 29526105 10.1021/acs.jpcb.8b02123 PMC6422163

[69] McCoy A. J., Grosse-Kunstleve R. W., Adams P. D., Winn M. D., Storoni L. C., & Read R. J. (2007). Phaser crystallographic software. Journal of Applied Crystallography, 40(Pt 4), 658–674. 19461840 10.1107/S0021889807021206 PMC2483472

[70] Mirkovic T., Ostroumov E. E., Anna J. M., van Grondelle R., Govindjee, & Scholes G. D. (2017). Light Absorption and Energy Transfer in the Antenna Complexes of Photosynthetic Organisms. Chemical Reviews, 117(2), 249–293. 27428615 10.1021/acs.chemrev.6b00002

[71] Moriarty N. W., Grosse-Kunstleve R. W., & Adams P. D. (2009). electronic Ligand Builder and Optimization Workbench (eLBOW): a tool for ligand coordinate and restraint generation. Acta Crystallographica. Section D, Biological Crystallography, 65(Pt 10), 1074–1080. 19770504 10.1107/S0907444909029436 PMC2748967

[72] Moser C. C., Sheehan M. M., Ennist N. M., Kodali G., Bialas C., Englander M. T., Discher B. M., & Dutton P. L. (2016). De Novo Construction of Redox Active Proteins. In Methods in Enzymology (pp. 365–388). 10.1016/bs.mie.2016.05.048 PMC5123760 27586341

[73] Myers J., Grothaus G., Narayanan S., & Onufriev A. (2006). A simple clustering algorithm can be accurate enough for use in calculations of pKs in macromolecules. Proteins, 63(4), 928–938. 16493626 10.1002/prot.20922

[74] Niwa S., Yu L.-J., Takeda K., Hirano Y., Kawakami T., Wang-Otomo Z.-Y., & Miki K. (2014). Structure of the LH1–RC complex from Thermochromatium tepidum at 3.0 Å. Nature, 508(7495), 228–232. 24670637 10.1038/nature13197

[75] Otwinowski Z., & Minor W. (1997). Processing of X-ray diffraction data collected in oscillation mode. Methods in Enzymology, 276, 307–326. 27754618 10.1016/S0076-6879(97)76066-X

[76] Pettersen E. F., Goddard T. D., Huang C. C., Couch G. S., Greenblatt D. M., Meng E. C., & Ferrin T. E. (2004). UCSF Chimera--a visualization system for exploratory research and analysis. Journal of Computational Chemistry, 25(13), 1605–1612. 15264254 10.1002/jcc.20084

[77] Pieper J., Rätsep M., Trostmann I., Schmitt F.-J., Theiss C., Paulsen H., Eichler H. J., Freiberg A., & Renger G. (2011). Excitonic energy level structure and pigment-protein interactions in the recombinant water-soluble chlorophyll protein. II. Spectral hole-burning experiments. The Journal of Physical Chemistry. B, 115(14), 4053–4065. 21417356 10.1021/jp111457t

[78] Pirro F., Schmidt N., Lincoff J., Widel Z. X., Polizzi N. F., Liu L., Therien M. J., Grabe M., Chino M., Lombardi A., & DeGrado W. F. (2020). Allosteric cooperation in a de novo-designed two-domain protein. Proceedings of the National Academy of Sciences, 117(52), 33246–33253. 10.1073/pnas.2017062117 PMC7776816 33318174

[79] Polizzi N. F., Wu Y., Lemmin T., Maxwell A. M., Zhang S.-Q., Rawson J., Beratan D. N., Therien M. J., & DeGrado W. F. (2017). De novo design of a hyperstable non-natural protein–ligand complex with sub-Å accuracy. In Nature Chemistry (Vol. 9, Issue 12, pp. 1157–1164). 10.1038/nchem.2846 PMC5859929 29168496

[80] Punjani A., & Fleet D. J. (2021). 3D variability analysis: Resolving continuous flexibility and discrete heterogeneity from single particle cryo-EM. Journal of Structural Biology, 213(2), 107702. 33582281 10.1016/j.jsb.2021.107702

[81] Punjani A., Rubinstein J. L., Fleet D. J., & Brubaker M. A. (2017). cryoSPARC: algorithms for rapid unsupervised cryo-EM structure determination. Nature Methods, 14(3), 290–296. 28165473 10.1038/nmeth.4169

[82] Qian P., Gardiner A. T., Šímová I., Naydenova K., Croll T. I., Jackson P. J., Nupur N., Kloz M., Čubáková P., Kuzma M., Zeng Y., Castro-Hartmann P., van Knippenberg B., Goldie K. N., Kaftan D., Hrouzek P., Hájek J., Agirre J., Siebert C. A., … Koblížek M. (2022). 2.4-Å structure of the double-ring *Gemmatimonas phototrophica* photosystem. Science Advances, 8(7), eabk3139. 35171663 10.1126/sciadv.abk3139 PMC8849296

[83] Qian P., Siebert C. A., Wang P., Canniffe D. P., & Hunter C. N. (2018). Cryo-EM structure of the Blastochloris viridis LH1–RC complex at 2.9 Å. Nature, 556(7700), 203–208. 29618818 10.1038/s41586-018-0014-5

[84] Qian P., Swainsbury D. J. K., Croll T. I., Salisbury J. H., Martin E. C., Jackson P. J., Hitchcock A., Castro-Hartmann P., Sader K., & Hunter C. N. (2021). Cryo-EM structure of the monomeric Rhodobacter sphaeroides RC–LH1 core complex at 2.5 Å. Biochemical Journal, 478(20), 3775–3790. 34590677 10.1042/BCJ20210631 PMC8589327

[85] Rabanal F., DeGrado W. F., & Dutton P. L. (1996). Toward the synthesis of a photosynthetic reaction center maquette: A cofacial porphyrin pair assembled between two subunits of a synthetic four-helix bundle multiheme protein. Journal of the American Chemical Society, 118(2), 473–474.

[86] Reppert M. (2023). Bioexcitons by Design: How Do We Get There? The Journal of Physical Chemistry. B. 10.1021/acs.jpcb.2c08787 36854126

[87] Roe D. R., & Brooks B. R. (2020). A protocol for preparing explicitly solvated systems for stable molecular dynamics simulations. The Journal of Chemical Physics, 153(5), 054123. 32770927 10.1063/5.0013849 PMC7413747

[88] Romero E., Novoderezhkin V. I., & van Grondelle R. (2017). Quantum design of photosynthesis for bio-inspired solar-energy conversion. Nature, 543(7645), 355–365. 28300093 10.1038/nature22012

[89] Schneidman-Duhovny D., Hammel M., Tainer J. A., & Sali A. (2013). Accurate SAXS profile computation and its assessment by contrast variation experiments. Biophysical Journal, 105(4), 962–974. 23972848 10.1016/j.bpj.2013.07.020 PMC3752106

[90] Schneidman-Duhovny D., Hammel M., Tainer J. A., & Sali A. (2016). FoXS, FoXSDock and MultiFoXS: Single-state and multi-state structural modeling of proteins and their complexes based on SAXS profiles. Nucleic Acids Research, 44(W1), W424–W429. 27151198 10.1093/nar/gkw389 PMC4987932

[91] Selikhanov G., Fufina T., Vasilieva L., Betzel C., & Gabdulkhakov A. (2020). Novel approaches for the lipid sponge phase crystallization of the Rhodobacter sphaeroides photosynthetic reaction center. IUCrJ, 7(Pt 6), 1084–1091. 10.1107/S2052252520012142 PMC7642779 33209319

[92] Sener M. K., Lu D., Ritz T., Park S., Fromme P., & Schulten K. (2002). Robustness and Optimality of Light Harvesting in Cyanobacterial Photosystem I. The Journal of Physical Chemistry. B, 106(32), 7948–7960.

[93] Şener M., Strümpfer J., Hsin J., Chandler D., Scheuring S., Hunter C. N., & Schulten K. (2011). Förster energy transfer theory as reflected in the structures of photosynthetic light-harvesting systems. Chemphyschem: A European Journal of Chemical Physics and Physical Chemistry, 12(3), 518–531. 21344591 10.1002/cphc.201000944 PMC3098534

[94] Sharma V. K., Mahammed A., Mizrahi A., Morales M., Fridman N., Gray H. B., & Gross Z. (2021). Dimeric Corrole Analogs of Chlorophyll Special Pairs. Journal of the American Chemical Society, 143(25), 9450–9460. 34014656 10.1021/jacs.1c02362 PMC8249354

[95] Sheffler W., Yang E. C., Dowling Q., Hsia Y., Fries C. N., Stanislaw J., Langowski M., Brandys M., Khmelinskaia A., King N. P., & Baker D. (2022). Fast and versatile sequence-independent protein docking for nanomaterials design using RPXDock. In bioRxiv (p. 2022.10.25.513641). 10.1101/2022.10.25.513641 PMC10237659 37216343

[96] Singharoy A., Maffeo C., Delgado-Magnero K. H., Swainsbury D. J. K., Sener M., Kleinekathöfer U., Vant J. W., Nguyen J., Hitchcock A., Isralewitz B., Teo I., Chandler D. E., Stone J. E., Phillips J. C., Pogorelov T. V., Mallus M. I., Chipot C., Luthey-Schulten Z., Tieleman D. P., … Schulten K. (2019). Atoms to Phenotypes: Molecular Design Principles of Cellular Energy Metabolism. Cell, 179(5), 1098–1111.e23. 31730852 10.1016/j.cell.2019.10.021 PMC7075482

[97] Srivastava A., Ahad S., Wat J. H., & Reppert M. (2021). Accurate prediction of mutation-induced frequency shifts in chlorophyll proteins with a simple electrostatic model. The Journal of Chemical Physics, 155(15), 151102. 34686046 10.1063/5.0064567

[98] Studier F. W. (2005). Protein production by auto-induction in high density shaking cultures. Protein Expression and Purification, 41(1), 207–234. 15915565 10.1016/j.pep.2005.01.016

[99] Suloway C., Pulokas J., Fellmann D., Cheng A., Guerra F., Quispe J., Stagg S., Potter C. S., & Carragher B. (2005). Automated molecular microscopy: the new Leginon system. Journal of Structural Biology, 151(1), 41–60. 15890530 10.1016/j.jsb.2005.03.010

[100] Svergun D. I., Richard S., Koch M. H., Sayers Z., Kuprin S., & Zaccai G. (1998). Protein hydration in solution: experimental observation by x-ray and neutron scattering. Proceedings of the National Academy of Sciences of the United States of America, 95(5), 2267–2272. 9482874 10.1073/pnas.95.5.2267 PMC19315

[101] Swainsbury D. J. K., Qian P., Jackson P. J., Faries K. M., Niedzwiedzki D. M., Martin E. C., Farmer D. A., Malone L. A., Thompson R. F., Ranson N. A., Canniffe D. P., Dickman M. J., Holten D., Kirmaier C., Hitchcock A., & Hunter C. N. (2021). Structures of *Rhodopseudomonas palustris* RC-LH1 complexes with open or closed quinone channels. Science Advances, 7(3), eabe2631. 33523887 10.1126/sciadv.abe2631 PMC7806223

[102] Tani K., Kanno R., Makino Y., Hall M., Takenouchi M., Imanishi M., Yu L.-J., Overmann J., Madigan M. T., Kimura Y., Mizoguchi A., Humbel B. M., & Wang-Otomo Z.-Y. (2020). Cryo-EM structure of a Ca2+-bound photosynthetic LH1-RC complex containing multiple αβ-polypeptides. Nature Communications, 11(1), 4955. 10.1038/s41467-020-18748-3 PMC7532537 33009385

[103] Taylor N., & Kassal I. (2019). Why are photosynthetic reaction centres dimeric? Chemical Science, 10(41), 9576–9585. 32055331 10.1039/c9sc03712h PMC6993572

[104] Tomasi J., Mennucci B., & Cammi R. (2005). Quantum mechanical continuum solvation models. Chemical Reviews, 105(8), 2999–3093. 16092826 10.1021/cr9904009

[105] Tramier M., Gautier I., Piolot T., Ravalet S., Kemnitz K., Coppey J., Durieux C., Mignotte V., & Coppey-Moisan M. (2002). Picosecond-hetero-FRET microscopy to probe protein-protein interactions in live cells. Biophysical Journal, 83(6), 3570–3577. 12496124 10.1016/S0006-3495(02)75357-5 PMC1302432

[106] van Amerongen H., Valkunas L., & van Grondelle R. (2000). Photosynthetic Excitons. World Scientific.

[107] Visschers R. W., Chang M. C., van Mourik F., Parkes-Loach P. S., Heller B. A., Loach P. A., & van Grondelle R. (1991). Fluorescence polarization and low-temperature absorption spectroscopy of a subunit form of light-harvesting complex I from purple photosynthetic bacteria. Biochemistry, 30(23), 5734–5742. 1904275 10.1021/bi00237a015

[108] Wahadoszamen M., Margalit I., Ara A. M., van Grondelle R., & Noy D. (2014). The role of charge-transfer states in energy transfer and dissipation within natural and artificial bacteriochlorophyll proteins. Nature Communications, 5, 5287. 10.1038/ncomms6287 PMC4255223 25342121

[109] Wasielewski M. R., Studier M. H., & Katz J. J. (1976). Covalently linked chlorophyll a dimer: A biomimetic model of special pair chlorophyll. Proceedings of the National Academy of Sciences of the United States of America, 73(12), 4282–4286. 16592367 10.1073/pnas.73.12.4282 PMC431433

[110] Weber G., & Teale F. W. J. (1957). Determination of the absolute quantum yield of fluorescent solutions. Transactions of the Faraday Society, 53(0), 646–655.

[111] Williams C. J., Headd J. J., Moriarty N. W., Prisant M. G., Videau L. L., Deis L. N., Verma V., Keedy D. A., Hintze B. J., Chen V. B., Jain S., Lewis S. M., Arendall W. B. 3rd, Snoeyink J., Adams P. D., Lovell S. C., Richardson J. S., & Richardson D. C. (2018). MolProbity: More and better reference data for improved all-atom structure validation. Protein Science: A Publication of the Protein Society, 27(1), 293–315. 29067766 10.1002/pro.3330 PMC5734394

[112] Winn M. D., Ballard C. C., Cowtan K. D., Dodson E. J., Emsley P., Evans P. R., Keegan R. M., Krissinel E. B., Leslie A. G. W., McCoy A., McNicholas S. J., Murshudov G. N., Pannu N. S., Potterton E. A., Powell H. R., Read R. J., Vagin A., & Wilson K. S. (2011). Overview of the CCP4 suite and current developments. Acta Crystallographica. Section D, Biological Crystallography, 67(Pt 4), 235–242. 21460441 10.1107/S0907444910045749 PMC3069738

[113] Wraight C. A., & Clayton R. K. (1974). The absolute quantum efficiency of bacteriochlorophyll photooxidation in reaction centres of Rhodopseudomonas spheroides. In Biochimica et Biophysica Acta (BBA) - Bioenergetics (Vol. 333, Issue 2, pp. 246–260). 10.1016/0005-2728(74)90009-7 19400037

[114] Yao S., Moyer A., Zheng Y., Shen Y., Meng X., Yuan C., Zhao Y., Yao H., Baker D., & Wu C. (2022). De novo design and directed folding of disulfide-bridged peptide heterodimers. Nature Communications, 13(1), 1–10. 10.1038/s41467-022-29210-x PMC8941120 35318337

[115] Yu L.-J., Suga M., Wang-Otomo Z.-Y., & Shen J.-R. (2018). Structure of photosynthetic LH1–RC supercomplex at 1.9 Å resolution. Nature, 556(7700), 209–213. 29618814 10.1038/s41586-018-0002-9

[116] Zhang Y., & Skolnick J. (2005). TM-align: a protein structure alignment algorithm based on the TM-score. Nucleic Acids Research, 33(7), 2302–2309. 15849316 10.1093/nar/gki524 PMC1084323

[117] Zheng S. Q., Palovcak E., Armache J.-P., Verba K. A., Cheng Y., & Agard D. A. (2017). MotionCor2: anisotropic correction of beam-induced motion for improved cryo-electron microscopy. Nature Methods, 14(4), 331–332. 28250466 10.1038/nmeth.4193 PMC5494038

